# Parent‐of‐Origin Effects in Childhood Asthma at Seven Years of Age

**DOI:** 10.1002/gepi.70007

**Published:** 2025-03-25

**Authors:** Yunsung Lee, Miriam Gjerdevik, Astanand Jugessur, Håkon Kristian Gjessing, Elizabeth Corfield, Alexandra Havdahl, Jennifer Ruth Harris, Maria Christine Magnus, Siri Eldevik Håberg, Per Magnus

**Affiliations:** ^1^ Centre for Fertility and Health Norwegian Institute of Public Health Oslo Norway; ^2^ Department of Computer Science, Electrical Engineering and Mathematical Sciences Western Norway University of Applied Sciences Bergen Norway; ^3^ Department of Global Public Health and Primary Care University of Bergen Bergen Norway; ^4^ PsychGen Centre for Genetic Epidemiology and Mental Health Norwegian Institute of Public Health Oslo Norway; ^5^ Nic Waals Institute Lovisenberg Diaconal Hospital Oslo Norway

**Keywords:** childhood asthma, genetics, GWAS, Haplin, Norwegian Mother, Father, and Child Cohort Study (MoBa), parent‐of‐origin

## Abstract

Childhood asthma is more common among children whose mothers have asthma than among those whose fathers have asthma. The reasons for this are unknown, and we hypothesize that genomic imprinting may partly explain this observation. Our aim is to assess parent‐of‐origin (PoO) effects on childhood asthma by analyzing SNP array genotype data from a large population‐based cohort. To estimate PoO effects in parent‐reported childhood asthma at 7 years of age, we fit a log‐linear model implemented in the HAPLIN R package to SNP array genotype data from 915 mother–father–child case triads, 603 mother–child case dyads, and 113 father–child case dyads participating in the Norwegian Mother, Father, and Child Cohort Study (MoBa). We found that alleles at two SNPs—rs3003214 and rs3003211—near the adenylosuccinate synthase 2 gene (*ADSS2* on chromosome 1q44) showed significant PoO effects at a false positive rate ≤ 0.05. The ratio of the effect of the maternally and paternally inherited G‐allele at rs3003214 was 1.68 (95% CI: 1.41–2.03, *p* value = 1.13E−08). Our results suggest PoO effects at the *ADSS2* gene, particularly the maternally inherited G‐allele at rs3003214, may contribute to the maternal effect in childhood asthma.

**Abbreviations**
FDR: false positive rate
MoBa: Norwegian Mother, Father, and Child Cohort Study
PoO: parent‐of‐origin
RRcf
: relative risk associated with paternally transmitted effect allele
RRcm
: relative risk associated with the maternally transmitted effect allele

## 1 Introduction

Asthma is characterized by variable respiratory obstruction and is the most common chronic disease in childhood. The heritability varies from 0.79 to 0.92 (Fagnani et al. 2008; Harris et al. 1997; Ullemar et al. 2016; van Beijsterveldt and Boomsma 2007). A repeated finding is that children more often have asthma if the mother has asthma than if the father has it (Jaakkola et al. 2001; Lim et al. 2010). However, it is still a mystery through which mechanisms this maternal effect is promoted. There are several genetic possibilities. One is mitochondrial inheritance, and another is the effect of maternal genes on the developing fetus during pregnancy, over and above the maternal genetic contribution in the fetal genome (maternal genetic effect). Environmental exposures to the mother may influence the fetus through epigenetic mechanisms (Harb et al. 2016; Potaczek et al. 2017). A third possibility is genomic imprinting, that allelic activity depends on the parental source of transmitted alleles. In epidemiological studies, one can observe this as parent‐of‐origin (PoO) differences (Ainsworth et al. 2011; Gjerdevik et al. 2018; Lawson et al. 2013; X. Yu et al. 2017; J. Y. Zhou et al. 2010; J. Y. Zhou et al. 2009). There is one large‐scale pedigree‐based study that reported loci with PoO effects on asthma and allergic rhinitis combined (Sarnowski et al. 2016). Several studies have found genetic evidence of imprinting in atopy, with a focus on the maternal inheritance of certain genes on chromosomes 11q, 6, and 14 and paternal allele clusters on chromosome 13, indicating a genetic component in allergic conditions (Cookson et al. 1992; Demenais et al. 2001; Nguyen and Liao 2015; Shirakawa et al. 1994; Strauch et al. 2001). Our aim is to estimate PoO effects for childhood asthma, potentially finding mechanisms for the maternal effect, by performing an agnostic analysis in SNP array genotype data from a large population‐based cohort, the Norwegian Mother, Father, and Child Cohort Study (MoBa).

## 2 Methods

### 2.1 Study Population

MoBa is a nationwide pregnancy cohort study in which approximately 95,000 mothers, 75,000 fathers, and 114,000 children were recruited from 1999 to 2009 across Norway (Magnus et al. 2016). The participation rate of invited pregnant women was 41%. Women could participate with more than one pregnancy during the recruitment period. The participants completed a series of questionnaires. Peripheral whole‐blood samples were collected from mothers and fathers around the 17th week of gestation and from the mothers (whole‐blood) and newborn children (cord blood) at delivery (Paltiel et al. 2014; Rønningen et al. 2006). DNA was extracted from blood samples after arrival at the biobank. Full details of genotyping, quality control, imputation, and post‐imputation quality control are described elsewhere (Corfield et al. 2022). A total of 207,569 unique individuals of European ancestry (76,577 children, 53,358 fathers, and 77,634 mothers) and 6,981,748 SNPs passed through the MoBaPsychGen pipeline. The imputed data were provided in PLINK 1.9 binary format, which supported only hard calls. Therefore, subsequent statistical analyses were based on the imputed data with hard calls.

### 2.2 Childhood Asthma at Seven Years of Age

When the children were 7 years old, a questionnaire focusing on allergies and asthma was sent to the parents (available at https://www.fhi.no/en/studies/moba/for-forskere-artikler/questionnaires-from-moba/#7yearolds). Mothers were asked whether the child had ever experienced asthma, whether this was confirmed/diagnosed by a doctor, and whether the child had experienced asthma symptoms or used medications for asthma in the past year. Current asthma was defined as doctor‐diagnosed asthma with symptoms and/or medication use during the past years. This resulted in 1723 children with and 34,995 children without asthma.

### 2.3 Statistical Analyses

To quantify SNP‐based PoO effects in childhood asthma, we fit log‐linear regression using the Haplin R package (version 7.3.0) (Gjessing and Lie 2006). The risk of childhood asthma given the genotypes of triads, that is, the disease penetrance, was modeled as follows: $\text{Pr}\left(D|M,F,C\right)=\text{Pr}\left(D|A_{i}A_{j},A_{k}A_{l},A_{j}A_{l}\right)=B∙\mathrm{RRcm}∙\mathrm{RRcf}∙{\mathrm{RR}}^{*}$, where M, F, and C are the, respectively, maternal, paternal, and offspring alleles, $A_{i}$ is a maternally non‐transmitted allele, $A_{j}$ is a maternally transmitted allele, $A_{k}$ is a paternally non‐transmitted allele, $A_{l}$ is a paternally transmitted allele, B is the baseline risk of asthma in the general population, RRcm is the relative risk associated with the maternally transmitted effect allele, RRcf is the relative risk associated with paternally transmitted effect allele, and ${\mathrm{RR}}^{*}$ is the relative risk for a double‐dose of the effect allele. By Bayes' theorem, the probability of the genotypes of triads given disease, *D*, is $\text{Pr}\left(M,F,\mathrm{C|D}\right)=\text{Pr}\left(D|M,F,C\right)\text{Pr}\left(M,F,C\right)/\text{Pr}(D)=B∙\mathrm{RRcm}∙\mathrm{RRcf}∙{\mathrm{RR}}^{*}∙p_{i}p_{j}p_{k}p_{l}/\text{Pr}(D)$, where $p_{i}$, $p_{j}$, $p_{k}$, and $p_{l}$ are the population allele frequencies for $A_{i}$, $A_{j}$, $A_{k}$, and $A_{l}$, respectively, and Pr(D) is the disease prevalence. The equation can be rewritten as $E\left(\mu_{\mathrm{ijkl}}\right)=\epsilon ∙\mathrm{RRcm}∙\mathrm{RRcf}∙{\mathrm{RR}}^{*}∙p_{i}p_{j}p_{k}p_{l}$ by multiplying both sides by the total number of triads given disease, where $\mu_{\mathrm{ijkl}}$ is the expected count of the triads given disease with certain alleles for $A_{i}$, $A_{j}$, $A_{k}$, and $A_{l}$ and ϵ is a normalizing constant. In our analyses, we set ${\mathrm{RR}}^{*}$ to 1, corresponding to a multiplicative dose–response model. For the current analyses, we considered SNPs with minor allele frequencies larger than 0.05. The significance of a PoO effect was determined by the *p* value of the relative risk ratio (i.e., RRcm */* RRcf).

Additionally, we considered SNPs that had moderate effects of maternally inherited alleles but no effects of paternally inherited alleles, and vice versa. This consideration arises because the significance of the relative risk ratio (RRcm/RRcf) is not sufficient to infer potential PoO effects. The RRcm/RRcf ratio can heighten when RRcm and RRcf are in opposite directions (e.g., RRcm is greater than 1.5, while RRcf is less than 0.7) but does not effectively capture cases where one term is significant, and the other remains nonsignificant.

As a sensitivity analysis, we also estimated haplotype‐based PoO effects in childhood asthma. A haplotype analysis should increase the chance of detecting a deleterious mutation if it lies within a region of SNPs. However, this analysis is subject to reduced statistical power and efficiency compared to a SNP‐based analysis (Sato et al. 2016). Thus, we restricted our haplotype‐based analyses to the genomic regions where SNPs with moderate to strong PoO effects were identified in the SNP‐based analysis, considering three neighboring SNPs at a time.

For the effects of maternal genotypes on childhood asthma, we added a parameter RRm to the abovementioned model for PoO effects, where RRm is the relative risk associated with the maternal effect allele.

To estimate the effects of the children's own genotypes on childhood asthma, we fit linear mixed regression to the genotype data on 1723 asthmatic children and 34,995 controls, as implemented in REGENIE (version 3.1.1) (Mbatchou et al. 2021) and SAIGE (version 1.1.1) (W. Zhou et al. 2018). The effect sizes were adjusted for batch and principal components 1–10. For REGENIE, we used parameter settings as follows: –bsize 200, –spa (saddle point approximation), and –pThresh 0.01. For SAIGE, we used the default parameters for the single‐variant association tests (it implements the saddle point approximation by default). For both software, we considered hard calls at loci with a minor allele frequency higher than 0.05. Both software programs were used for the following reasons. REGENIE is highly efficient at performing GWASs quickly, making it suitable for analyzing large datasets with speed and scalability (Mbatchou et al. 2021). However, it can produce overly conservative results, especially in datasets with large families or highly correlated data such as MoBa (Gurinovich et al. 2022). On the other hand, SAIGE is noted for its robust control of type 1 error rates and performs well in small and correlated data settings (W. Zhou et al. 2018). However, SAIGE is less efficient in handling missing genotype data (Gurinovich et al. 2022). Using both tools allowed us to ensure a comprehensive and reliable analysis.

## 3 Results

### 3.1 PoO Effects in Childhood Asthma

To estimate PoO effects, we only included asthmatic children for whom information on their parent's genotypes as well as their own were available. This resulted in 915 mother–father–child triads, 603 mother–child dyads, and 113 father–child dyads (*n* = 1631, Figure 1). Alleles at two SNPs—rs3003214 and rs3003211—near the adenylosuccinate synthase 2 gene (*ADSS2* on chromosome 1q44) showed significant PoO effects in childhood asthma at a false positive rate (FDR) less than or equal to 0.05 (Figure 2 and Table 1). When the effect allele G at rs3003214 was maternally inherited, it was associated with an increased relative risk of childhood asthma (RRcm = 1.37, 95% CI: 1.19–1.58, *p* value = 1.71E−05). By contrast, the same allele was associated with a reduced relative risk of childhood asthma when inherited from the father (RRcf = 0.81, 95% CI: 0.70–0.94, *p* value = 4.65E−03). The ratio of the effect of the maternally and paternally inherited G‐allele at rs3003214 was 1.68 (95% CI: 1.41–2.03, *p* value = 1.13E−08). A similar interpretation applies to rs3003211 because this SNP was in high linkage disequilibrium (R^2^ = 0.9934) with rs3003214.

**Figure 1:**
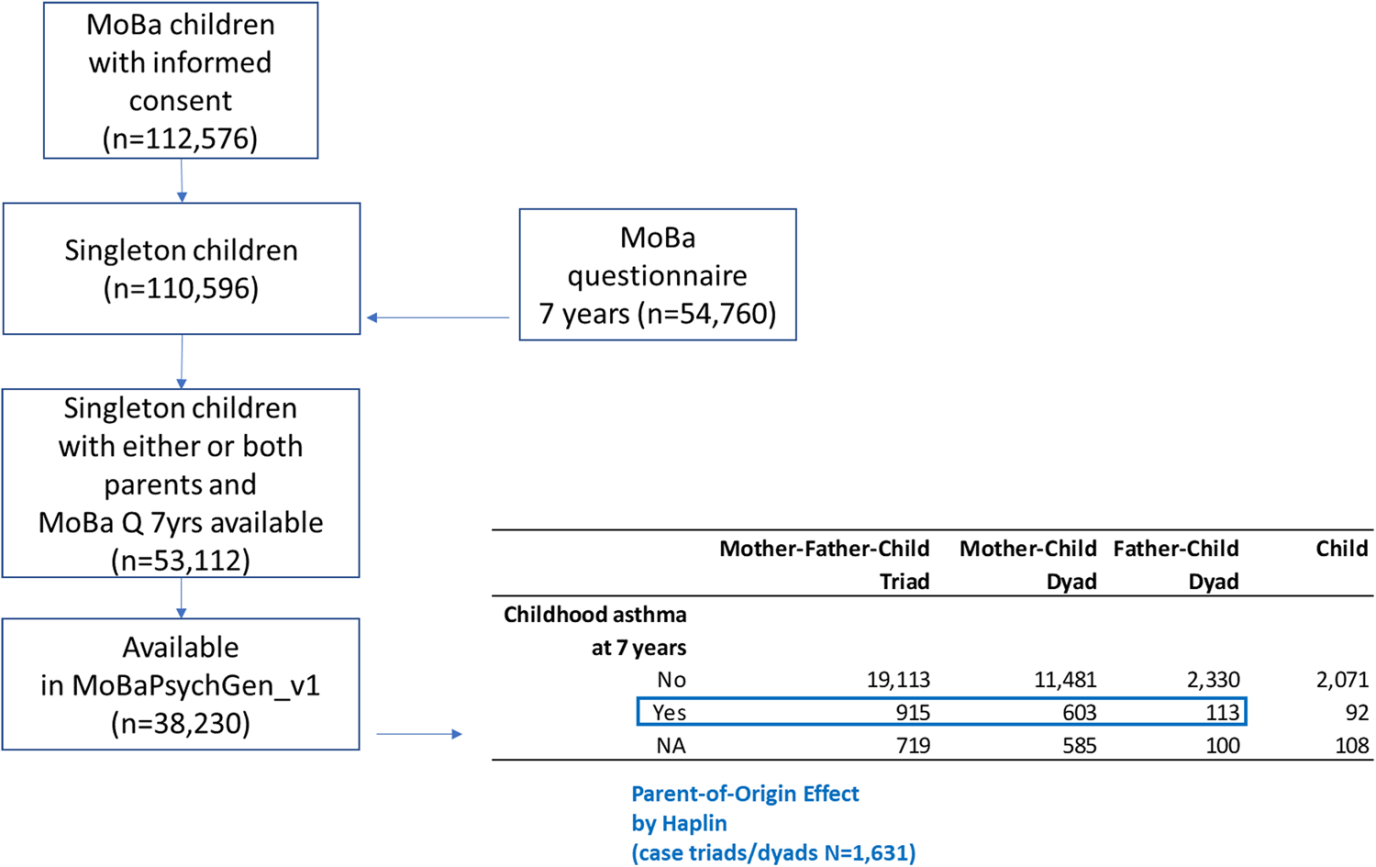
Overview of the sampling scheme.

**Figure 2:**
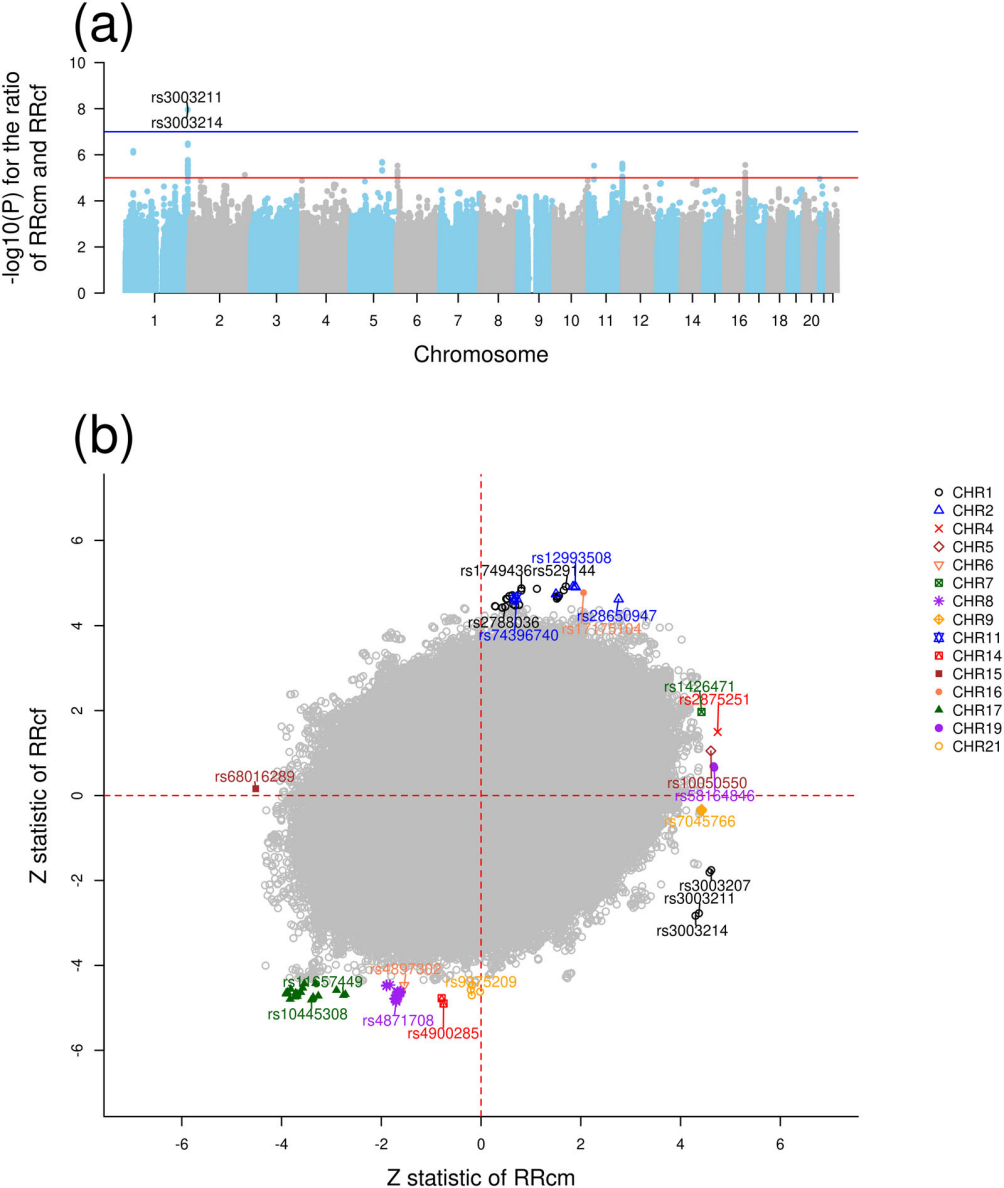
Parent‐of‐origin effects in childhood asthma. (a) Manhattan plot showing the significance of the ratio RRcm/RRcf across the whole genome. The red line refers to −log(1E−05)=5, while the blue line refers to −log(1E−07)=7. These lines do not indicate any statistical significance but were set to improve the visibility of this plot. (b) Comparison between *Z* statistic for the risk of childhood asthma by maternally inherited alleles (RRcm) and that by paternally inherited alleles (RRcf) at each SNP. *Z* statistic was calculated as the logarithm of either RRcm or RRcf divided by its standard error. The scatter plot displays, in different colors, the two SNPs detected in (a) and other SNPs where the *p* value of either relative risk by maternally or paternally inherited alleles was below 10E−05. The SNPs with RSID numbers were selected after linkage disequilibrium‐based clumping.

**Table 1:** SNPs with parent‐of‐origin effects in childhood asthma.

SNP^a^	CHR	POS^b^	A1^c^	A2^d^	MAF^e^	HWE *p* value^f^	Gene names^g^	RRcm (95% CI)^h^	RRcm *p* value^h^	RRcf (95% CI)^i^	RRcf *p* value^i^	RRcm/RRcf (95% CI)^j^	RRcm/RRcf *p* value^j^
rs1749436	1	43632353	C	G	0.414	0.345	*EBNA1BP2*	1.06 (0.92, 1.23)	4.18E−01	1.39 (1.21, 1.59)	1.11E−06	0.77 (0.64, 0.91)	2.41E−03
rs2788036	1	53770124	T	C	0.360	0.371	*LRP8*	1.04 (0.89, 1.2)	6.27E−01	1.35 (1.19, 1.55)	8.99E−06	0.77 (0.64, 0.91)	3.03E−03
rs529144	1	182405493	G	T	0.171	0.349		1.17 (0.97, 1.38)	8.95E−02	1.49 (1.27, 1.75)	8.79E−07	0.78 (0.64, 0.95)	1.24E−02
rs3003214*	1	244605036	G	A	0.324	0.211	*ADSS2*	1.37 (1.19, 1.58)	1.71E−05	0.81 (0.7, 0.94)	4.65E−03	1.68 (1.41, 2.03)	1.13E−08
rs3003211*	1	244612984	T	C	0.326	0.227	*ADSS2*	1.37 (1.19, 1.58)	1.25E−05	0.81 (0.7, 0.94)	5.55E−03	1.69 (1.41, 2.01)	1.09E−08
rs3003207*	1	244631821	C	T	0.397	0.207	*C1orf101*	1.4 (1.21, 1.61)	3.95E−06	0.88 (0.77, 1.02)	7.89E−02	1.58 (1.32, 1.88)	3.75E−07
rs28650947	2	55073797	C	A	0.056	0.766	*EML6*	1.43 (1.11, 1.85)	5.81E−03	1.76 (1.39, 2.25)	3.92E−06	0.81 (0.63, 1.05)	1.08E−01
rs12993508	2	125310284	A	T	0.266	0.601	*CNTNAP5*	1.16 (0.99, 1.35)	5.91E−02	1.42 (1.23, 1.63)	8.85E−07	0.82 (0.69, 0.97)	2.26E−02
rs2875251*	4	23566880	T	C	0.204	0.451	*PPARGC1A*	1.46 (1.25, 1.71)	2.10E−06	1.13 (0.96, 1.32)	1.34E−01	1.29 (1.08, 1.55)	4.64E−03
rs10050550*	5	26121701	G	C	0.067	0.400	*CDH9*	1.7 (1.36, 2.13)	4.06E−06	1.14 (0.89, 1.46)	2.93E−01	1.49 (1.16, 1.91)	1.85E−03
rs4897302	6	123886231	C	T	0.440	0.663	*TRDN*	0.89 (0.77, 1.03)	1.23E−01	0.73 (0.64, 0.84)	7.88E−06	1.22 (1.02, 1.45)	3.00E−02
rs1426471*	7	135942399	C	T	0.128	0.561		1.5 (1.25, 1.8)	9.87E−06	1.2 (1, 1.45)	4.90E−02	1.25 (1.02, 1.53)	3.01E−02
rs4871708	8	127432057	G	T	0.212	0.828		0.87 (0.73, 1.02)	8.90E−02	0.65 (0.55, 0.78)	1.22E−06	1.33 (1.07, 1.63)	8.21E−03
rs7045766*	9	27224358	T	A	0.399	0.411	*TEK*	1.37 (1.19, 1.58)	9.78E−06	0.98 (0.86, 1.12)	7.43E−01	1.41 (1.18, 1.66)	8.99E−05
rs74396740	11	84461408	T	C	0.072	0.698	*DLG2*	1.09 (0.86, 1.4)	4.76E−01	1.67 (1.35, 2.08)	3.13E−06	0.65 (0.51, 0.84)	8.12E−04
rs4900285	14	96094706	C	T	0.503	0.370		0.95 (0.82, 1.09)	4.52E−01	0.72 (0.63, 0.82)	9.75E−07	1.32 (1.11, 1.56)	1.72E−03
rs68016289*	15	61292152	T	C	0.139	0.880	*RORA*	0.6 (0.48, 0.75)	6.26E−06	1.01 (0.85, 1.22)	8.72E−01	0.59 (0.46, 0.76)	3.35E−05
rs17175104	16	54273895	G	A	0.063	0.197	*IRX3*	1.3 (1.01, 1.67)	3.99E−02	1.74 (1.39, 2.2)	1.85E−06	0.74 (0.58, 0.96)	2.11E−02
rs10445308	17	37938047	C	T	0.488	0.774	*IKZF3*	0.78 (0.67, 0.9)	6.68E−04	0.72 (0.63, 0.83)	1.50E−06	1.08 (0.9, 1.28)	3.99E−01
rs11657449	17	38057841	G	C	0.321	0.275	*GSDMB*	0.81 (0.69, 0.94)	5.75E−03	0.7 (0.6, 0.81)	2.53E−06	1.16 (0.95, 1.4)	1.41E−01
rs58164846*	19	41140115	G	A	0.191	0.009	*LTBP4*	1.46 (1.25, 1.72)	2.95E−06	1.05 (0.9, 1.25)	5.11E−01	1.39 (1.15, 1.67)	6.60E−04
rs9975209	21	35549135	C	T	0.517	0.509		0.99 (0.85, 1.14)	8.53E−01	0.73 (0.64, 0.83)	2.55E−06	1.36 (1.14, 1.61)	6.32E−04

^a^ SNPs with stronger effects of maternally inherited alleles than that of paternally inherited alleles are marked with asterisk.

^b^ Nucleotide positions are according to the Genome Reference Consortium Human Build 37 (GRCh37).

^c^ Reference allele.

^d^ Effect allele.

^e^ Minor allele frequency.

^f^ Hardy–Weinberg equilibrium test.

^g^ Gene names were retrieved from the Ensembl GRCh37. When a SNP is not located within a gene, the gene nearest to the SNP is listed instead.

^h^ Relative risk of childhood asthma due to maternally inherited alleles.

^i^ Relative risk of childhood asthma due to paternally inherited alleles.

^j^ Ratio of the relative risks due to the maternally and paternally inherited alleles.

Further, we focused on SNPs that had moderate effects of maternally inherited alleles but no effects of paternally inherited alleles, and vice versa (Figure 2 and Table 1). We chose SNPs that had a *p* value less than 10E−05 for either RRcm or RRcf and performed a linkage disequilibrium‐based clumping to obtain a set of independent SNPs. The maternally transmitted allele T at rs2788036 showed no effect on the risk of childhood asthma (RRcm = 1.04, 95% CI: 0.89–1.2, *p* value = 6.27E−01). However, the paternally transmitted allele increased the risk of childhood asthma (RRcf = 1.35, 95% CI: 1.19–1.55, *p* value = 8.99E−06). Regional plots around the SNPs listed in Table 1 are provided in Figure S1, Supporting Information File 1, and the summary statistics can be found in Supporting Information File 2.

We conducted sensitivity analyses, focusing on the 22 SNPs listed in Table 1. These analyses included: (1) a haplotype‐based analysis (Supporting Information File 3), (2) an SNP‐based analysis conducted separately for boys (*n* = 986) and girls (*n* = 645) (Table S1 and S2, Supporting Information File 1), and (3) an SNP‐based analysis including one child from each triad, that is, no siblings included in the data (*n* = 1604, Table S3, Supporting Information File 1). The analysis of haplotypes suggested that the haplotypes that contained rs3003214 (e.g., haplotype T‐A‐A, RRcm/RRcf = 1.56, *p* value = 2.05E−06, where the reference was T‐A‐G at rs3003215‐rs3006001‐rs3003214) and rs3003211 (e.g., haplotype G‐C‐T, RRcm/RRcf = 1.49, *p* value = 6.31E−05, where the reference was A‐T‐C at rs3003212‐rs3003211‐rs6667957) might have PoO effects. The SNP‐based analysis for each sex produced almost identical results as those reported in Table 1 but showed higher *p* values due to smaller sample sizes. The SNP‐based analysis, including one child from each triad, generated a consistent result as that in Table 1. We detected 25 full‐sibling pairs and 1 full‐sibling triad.

### 3.2 Effects of Maternal Genotypes on the Risk of Childhood Asthma

As an additional analysis, we examined the mother's genetic effects on her children's asthma with adjustment for the PoO effect. We did not find any SNPs showing maternal effects at FDR less than or equal to 0.05 but spotted moderate maternal effects at SNPs such as rs10841054 (*p* value = 3.94E−07) and rs10842885 (*p* value = 7.79E−07) that were located near *PIK3C2G* on chromosome 12p12.3 and *STK38L* on chromosome 12p11.23, respectively (Figure S2, Supporting Information File 1).

### 3.3 GWAS of Children's Own Genotype and Risk of Childhood Asthma

As shown and discussed in Supporting Information File 1, we first performed a genome‐wide association study (GWAS) of children's own genotype and childhood asthma. This analysis was based on 1723 asthmatic children at 7 years of age (defined as cases) and 34995 control children. We found strong associations between childhood asthma and variants near gasdermin B (*GSDMB*) and zona pellucida‐binding protein 2 (*ZPBP2*) on chromosome 17q21.1 (e.g., rs4795399‐T, odds ratio = 1.43, *p* value = 1.61E−23), which is consistent with previous studies (Moffatt et al. 2010; Pividori et al. 2019; Verlaan et al. 2009) (Table S4 and Figure S3, Supporting Information File 1). Additionally, we assessed the association between rs4795399‐T and childhood asthma in boys and girls separately and found no significant difference between the sexes (odds ratio for boys = 1.38 and for girls = 1.48, *p* value for the difference = 0.31).

## 4 Discussion

When searching for PoO effects in childhood asthma across the whole genome, we found that the effect alleles at two SNPs, rs3003214 and rs3003211, near the gene for adenylosuccinate synthase 2 (*ADSS2* on chromosome 1q44) were significantly associated with childhood asthma in opposite directions depending on the parental origin of the alleles. At 20 SNPs, the alleles transmitted from one parent showed moderate effects on the risk of childhood asthma, while the same alleles showed no effect when transmitted from the other parent.

Our PoO analyses identified moderate to strong associations with several genes previously reported to be associated with asthma. This includes PPARG coactivator 1 α (*PPARGC1A* on chr 4p15.2), Discs large MAGUK scaffold protein 2 (*DLG2* on chr 11q14.1), RAR‐related orphan receptor A (*RORA* on 15q22.2), and *GSDMB* (on 17q21.1) (highlighted in bold in Table 2). *PPARGC1A* encodes a transcriptional coactivator that regulates mitochondrial function and glucose metabolism (Y. Li et al. 2023). Of specific relevance for asthma and obstructive pulmonary disease, mitochondrial mass and oxygen consumption were shown to be higher in the bronchial smooth muscle from asthmatic individuals compared to that from chronic obstructive pulmonary disease (COPD) patients and controls (Sun et al. 2019; Trian et al. 2007). This process is likely to involve enhanced mitochondrial biogenesis through the upregulation of *PPARGC1A* (Callender et al. 2021; Trian et al. 2007).

**Table 2:** Synopsis of the top gene findings in the PoO and children's own genotypic effects on childhood asthma.

Gene ID^a^	Chr	Gene name	OMIM#^b^	Comments^c^	References
*ADSS2*	1q44	Adenylosuccinate synthase 2	103060	The protein product of this gene is an enzyme that catalyzes the conversion of inosine monophosphate to adenosine monophosphate. According to the Gene Cards database (https://www.genecards.org/), *ADSS2* “plays an important role in the *de novo* pathway and in the salvage pathway of purine nucleotide biosynthesis.” We did not find any links between this gene and asthma in the literature.	Powell et al. (1992)
*LRP8*	1p32.3	LDL receptor‐related protein 8	602600	This gene encodes a member of the low‐density lipoprotein receptor (LDLR) family. It is involved in lysosomal degradation and the migration of neurons during development. It is also involved in the cholesterol transport protein APOE. Some evidence indicates that variants in this gene are associated with myocardial infarction and coronary artery disease. However, we did not find any links between *LRP8* and asthma in the literature.	Shen et al. (2014); Shen et al. (2007)
*EML6*	2p16.1	Echinoderm microtubule‐associated protein‐like 6	NA	Only sparse information is available on this gene. EML6 is predicted to be located in the cytoplasm and microtubule. Its predicted function is to facilitate microtubule‐binding activity. Variants in this gene have been reported in GWASs of several traits, including refractive astigmatism, BMI, and economic and political preferences. We found no links between *LRP8* and asthma in the literature.	Benjamin et al. (2012); Q. Li et al. (2015)
***PPARGC1A***	4p15.2	PPARG coactivator 1 α	604517	This gene encodes a transcriptional coactivator that regulates genes involved in energy metabolism. PPARGC1A regulates mitochondrial function and glucose metabolism and plays an important role in cancer progression. With relevance to asthma and obstructive pulmonary disease specifically, mitochondrial mass and oxygen consumption were found to be higher in the bronchial smooth muscle from asthmatic individuals. This process involves enhanced mitochondrial biogenesis through the upregulation of *PPARGC1A*. In addition, rare variants in *PPARGC1A* have been associated with a risk of early‐onset and familial Parkinson's disease.	Callender et al. (2021); L. Z. Li et al. (2022); Y. Li et al. (2023); Sun et al. (2019); Trian et al. (2007)
*CDH9*	5p14.1	Cadherin 9	609974	This gene encodes a type II member of the cadherin superfamily of cell–cell adhesion molecules. Cadherins are calcium‐dependent transmembrane adhesion proteins that mediate cell–cell adhesion by binding to identical cadherin molecules on other cells through the process of homophilic binding. We did not find any links between this gene and asthma in the literature.	Tepass et al. 2000
*TEK*	9p21.2	TEK receptor tyrosine kinase	600221	This gene encodes a receptor belonging to the protein tyrosine kinase Tie2 family. Mutations in *TEK* are associated with inherited venous malformations of the skin and mucous membranes. Variation in TEK was not found to be associated with asthma but with allergic conjunctivitis.	Fodor et al. (2018)
***DLG2***	11q14.1	Discs large MAGUK scaffold protein 2	603583	The protein product of this gene is a member of the membrane‐associated guanylate kinase (MAGUK) family. A genome‐wide *interaction* analysis of air pollution exposure and childhood asthma showed that interactions with three genes, *ADCY2*, *B4GALT5*, and *DLG2*, were important for asthma development.	Gref et al. (2017)
***RORA***	15q22.2	RAR‐related orphan receptor A	600825	*RORA* is a known candidate gene for asthma. It encodes a member of the nuclear hormone receptor 1 (NR1) subfamily and is involved in a wide variety of processes, including the regulation of inflammation, apoptosis, autophagy, oxidative stress, etc. Variants in this gene have been associated with a reduced risk of allergic rhinitis. RORA may also play a critical regulatory role in T helper‐2 (Th2) cells which are involved in the pathogenesis of allergic asthma. Moreover, an interaction between *RORA* and another gene called neuropeptide S Receptor 1 (*NPSR1*) has been reported to be involved in nocturnal asthma.	Chen et al. (2023); Gaertner et al. (2019); Laubhahn et al. (2022); Lee et al. (2021); Lian et al. (2022); Lima et al. (2019)
*IRX3*	16q12.2	Iroquois homeobox 3	612985	*IRX3* belongs to the Iroquois homeobox gene family which is involved in pattern formation of vertebrate embryos. This gene has also been implicated in the development of obesity and fatty liver disease. We did not find any links between this gene and asthma in the literature.	Bellefroid (1998); de Araújo and Velloso (2020); Ma et al. (2022); Nagel (2023)
***GSDMB***	17q21.1	Gasdermin B	611221	This gene encodes a member of the gasdermin‐domain‐containing family of proteins that are involved in a wide range of cellular processes, including cell differentiation, coagulation, inflammation, and tumor development. They are also involved in pyroptosis and in regulating antimicrobial response. *GSDMB* lies within a region spanning chr 17q12‐q21 that is the most replicated genetic locus for childhood‐onset asthma. The mechanism underlying the association is thought to involve the regulation of *GSDMB* expression in airway epithelial cells. Another known asthma gene, *CDHR3*, appears to interact with *GSDMB*. Variants in *GSDMB* have also been found to be associated with chronic rhinosinusitis, an inflammatory condition affecting the nasal and sinus mucosa and that shows a high co‐occurrence with asthma. eQTL analysis based on gene‐expression data showed that multiple SNPs in *GSDMB* were associated with the severity of asthma. Another eQTL fine‐mapping analysis of the 17q12‐21 locus showed that *GSDMB* is the leading candidate gene for asthma susceptibility both in African Americans and in European Americans.	Eliasen et al. (2022); X. Li et al. (2021); Ntontsi et al. (2021); Ober et al. (2020); Schoettler et al. (2022); Zack et al. (2021); Zou et al. (2021)
***ZPBP2***	17q21.1	Zona pellucida‐binding protein 2	608499	This gene is predicted to be involved in the assembly of a cap‐like structure covering the head of a sperm (acrosome). It is also thought to be involved in the process where the sperm binds to the extracellular matrix (zona pellucida) surrounding an oocyte. The 17q12‐q21 haploblock described for *GSDMB* also encompasses *ZPBP2*. Risk variants in this core region containing the genes *IKZF3*, *ZPBP2*, *GSDMB*, *ORMDL3*, and *GSDMA* (in this order) were associated with multi‐trigger wheeze, which is an initial symptom of asthma. When this gene is knocked out in mice, airway hypersensitivity and lung lipid metabolism are affected. A meta‐analysis of GWASs of asthma in Puerto Ricans showed that SNPs in the region encompassing *ZPBP2* were significantly associated with asthma. T cells are one of the main types of cells affected by variants at 17q21, and this locus also seems to interact with early‐life environmental exposures.	Blekic et al. (2013); Kanagaratham et al. (2018); Laubhahn et al. (2022); Naumova et al. (2013); Schmiedel et al. (2016); Yan et al. (2017)
*LTBP4*	19q13.2	Latent transforming growth factor β‐binding protein 4	604710	This gene encodes a protein that binds to transforming growth factor β (TGFB) when it is secreted and transported into the extracellular matrix. We did not find any links between this gene and asthma in the literature.	Oklu and Hesketh 2000

^a^ Genes that have shown strong associations with asthma in previously published studies are highlighted in bold.

^b^ These numbers are according to the online catalog of human genes and genetic disorders, OMIM (https://www.omim.org/). NA, not available.

^c^ Information on the genes was collated from various sources: (1) *Entrez Gene* (https://www.ncbi.nlm.nih.gov/gene), (2) *Gene Cards* (https://www.genecards.org/), (3) *OMIM* (https://www.omim.org/), and (4) the included references.

The next top gene associated with PoO effects in our list, *DLG2*, encodes a member of the membrane‐associated guanylate kinase (MAGUK) family. A genome‐wide *interaction* analysis of air pollution exposure and childhood asthma showed interactions with three genes, *ADCY2*, *B4GALT5*, and *DLG2*, which have been found to be important for the development of asthma (Gref et al. 2017). Similarly, *RORA* has an established role as a risk gene for asthma. This gene encodes a member of the nuclear hormone receptor 1 (NR1) subfamily known to be involved in a wide array of biological processes, including the regulation of inflammation, apoptosis, autophagy, and oxidative stress, among others (reviewed in [Chen et al. 2023]). Genetic variants in *RORA* have also been associated with risk of allergic rhinitis and multi‐trigger wheeze (Laubhahn et al. 2022; Lian et al. 2022), with asthma and allergy markers in an admixed population (Lima et al. 2019), and with the regulation of T helper‐2 (Th2) cells that are closely involved in the pathogenesis of allergic asthma (Lee et al. 2021). Finally, an interaction between *RORA* and another gene, neuropeptide S Receptor 1 (*NPSR1*), appears to play a significant role in nocturnal asthma (Gaertner et al. 2019).

The last gene on our list, *GSDMB*, is also a recognized gene for childhood asthma (Schoettler et al. 2022). It encodes a member of the gasdermin domain containing a family of proteins known to play critical roles in a wide variety of cellular processes, including cellular differentiation, coagulation, inflammation, and tumor development (Ntontsi et al. 2021; Zou et al. 2021). Members of this gene family are also involved in the regulation of antimicrobial response and pyroptosis—a highly inflammatory form of programmed cell death that occurs postinfection with intracellular pathogens (P. Yu et al. 2021). Like *ZPBP2* that was identified in our GWAS of children's own genotypes and childhood asthma, *GSDMB* also lies within the same core region on 17q12‐q21 that is strongly linked to childhood‐onset asthma. The mechanism behind the association is thought to involve the regulation of *GSDMB* expression in airway epithelial cells. A genome‐wide study of early and severe childhood asthma pointed to a significant interaction between another known gene for asthma, *CDHR3*, and *GSDMB* (Eliasen et al. 2022). Further, a SNP in *GSDMB* (rs7216389) was also found to be associated with chronic rhinosinusitis, an inflammatory condition affecting the nasal and sinus mucosa, correlating strongly with the occurrence of asthma (Zack et al. 2021). Additionally, eQTL analysis using gene‐expression data revealed multiple SNPs in *GSDMB* that were associated with the severity of asthma, exacerbations, and antiviral pathways (X. Li et al. 2021). Another eQTL fine‐mapping analysis of the 17q12‐21‐haploblock region showed that *GSDMB* was the leading candidate gene for asthma susceptibility in both African Americans and European Americans (Ober et al. 2020).

Reassuringly, the results of our GWAS of children's own genotypes and childhood asthma (Table S4 and Figure S3, Supporting Information File 1) confirmed the strong associations previously reported between childhood asthma and chromosomal region 17q12‐q21 (Blekic et al. 2013; Laubhahn et al. 2022; Naumova et al. 2013; Schmiedel et al. 2016). As previously mentioned, this region houses a handful of genes, for example, *IKZF3*, *ZPBP2*, *GSDMB*, *ORMDL3*, and *GSDMA* (in this order), that have shown strong associations with asthma in several studies. This core region has also been reported to interact with early‐life environmental exposures in conjunction with childhood asthma (Blekic et al. 2013). Lastly, risk variants in genes within the 17q12‐q21‐haploblock have been associated with multi‐trigger wheeze, which is an initial symptom of asthma (Laubhahn et al. 2022). However, our findings did not reveal any associations between the 6p.21 region (*HLA‐B*, *HLA‐C*, *HLA‐DQA1*, and *HLA‐DQB*) and childhood asthma, despite previous reports suggesting such an association (Pividori et al. 2019). One possible explanation for this discrepancy could be differences in statistical power. Our study included 1723 asthmatic cases, while Pividori et al. (2019) included 9433 cases. Furthermore, heterogeneity in case selection might have also contributed to some of the observed differences. For example, Pividori et al. (2019) defined childhood asthma as onset before 12 years of age, while in our study, the onset was considered before 7 years of age.

Our GWAS of children's own genotypes and childhood asthma identified associations in *ZPBP2* (on chromosome 17q21.1), a gene predicted to be involved in the assembly of the cap‐like structure covering the head of a sperm (acrosome) and in the process through which a sperm binds to the extracellular matrix (zona pellucida) surrounding an oocyte. The strong link between *ZPBP2* and asthma has also been demonstrated in murine models. For example, knocking out *Zpbp2* in mice affects airway hypersensitivity and lung lipid metabolism (Kanagaratham et al. 2018). Additional evidence from human studies stems from a meta‐analysis of GWAS studies of asthma in a Puerto Rican population, which revealed a significant association between SNPs in the region encompassing *ZPBP2* and asthma (Yan et al. 2017). A potential mechanism for these associations is the observation that T cells were one of the main cell types affected by variants at 17q21 (Callender et al. 2021; Schmiedel et al. 2016).

However, our results on PoO effects were not consistent with those reported by Sarnowski et al. (2016). The two SNPs, rs10009104 and rs7687115, identified by Sarnowski et al. (2016) as having PoO effects on asthma and allergic rhinitis, showed RRcm/RRcf = 1.10 (*p* value = 0.32) and RRcm/RRcf = 0.99 (*p* value = 0.91) in our analysis. This discrepancy may be due to the different phenotype definitions. While our study focuses on childhood asthma alone, Sarnowski et al. (2016) included both asthma and allergic rhinitis.

Genomic imprinting is a potential mechanism behind maternal or PoO effects. However, there are other genetic mechanisms, such as maternal genetic effects (“genetic nurture” [Kong et al. 2018]) beyond the maternal contribution to the fetal genome and the influence of mitochondrial genes. Additionally, several maternally transmitted environmental exposures could also potentially influence asthma risk. In addition to replication in other asthma cohorts, our PoO findings need more in‐depth molecular genetic studies to confirm that genomic imprinting is indeed present.

As a limitation, firstly, the study population comprised individuals of European ancestry only. Secondly, our findings were not replicated in other independent cohorts. We have not yet found large‐scale cohorts with a sufficiently large number of genotyped triads or dyads. Thirdly, the asthmatic triads and dyads in this study were identified based on the self‐reported questionnaire, potentially including both persistent and transient cases (e.g., wheezing due to infections). However, the physician‐confirmed asthma diagnosis at age 7 likely excluded most transient wheezing disorders (Piippo‐Savolainen and Korppi 2008). We acknowledge potential improvement in defining asthmatic children, for example, by combining childhood asthma and allergic rhinitis or using more refined data from nationwide prescription records.

Given that the overall participation rate in MoBa was 41%, it is valid to consider if selection bias could have impacted the validity of our findings. A previous publication reported that MoBa well represented all Norwegian births but slightly underrepresented certain minor groups; this included young (at age < 25 years), single or smoking mothers, and those who experienced stillbirths and neonatal deaths (Nilsen et al. 2009). However, although the prevalence of certain characteristics differed between participants and nonparticipants, that study showed that exposure–outcome associations remained unbiased. If the variables characterizing these groups were to influence genomic variants and childhood asthma, this selection bias could lead to confounding in a conventional case–control design (Starks et al. 2009). However, the log‐linear model by HAPLIN protects against such confounding when fit to case triads and dyads (Gjessing and Lie 2006). This is because the HAPLIN model is based on comparing transmitted and non‐transmitted alleles from the same source population (the parents), which are likely to be equally affected by confounding. When considering environmental exposures influencing childhood asthma, it is important to note that further research is needed to explore potential interaction effects between environmental factors, for example, maternal smoking and PoO alleles (Gjerdevik et al. 2018).

In conclusion, our current analyses point to potential PoO effects in childhood asthma. Identifying PoO effects is critical because they can help illuminate the roles of genomic imprinting and maternal/paternal effects in disease pathogenesis, offering new insights into the mechanisms of inheritance and potentially aiding the development of more targeted therapeutic interventions.

## Author Contributions

Per Magnus and Håkon Kristian Gjessing designed the study. Yunsung Lee conducted statistical analyses under the supervision of Miriam Gjerdevik and Håkon Kristian Gjessing. Yunsung Lee, Per Magnus, and Astanand Jugessur drafted the manuscript. Elizabeth Corfield and Alexandra Havdahl performed quality control of the genotype data using the pipeline of *MoBaPsychGen‐V1*. Astanand Jugessur, Jennifer Ruth Harris, Maria Christine Magnus, and Siri Eldevik Håberg interpreted the findings. Siri Eldevik Håberg and Per Magnus acquired funding and administered the study. All the authors were involved in drafting the manuscript to its current form.

## Ethics Statement

This study was approved by the Norwegian Regional Committee for Medical Research (REK) South‐East C (reference number 1). Data collection by MoBa was carried out in accordance with the Norwegian Data Protection Agency and after approval from REK was secured.

## Consent

Written consents were obtained from the MoBa participants.

## Conflicts of Interest

The authors declare no conflicts of interest.

## Supporting information

Supporting information.

Supporting information.

Supporting information.

Supporting information.

## Data Availability

Access to the genetic datasets can be obtained by applying to the Norwegian Institute of Public Health (NIPH; http://www.fhi.no/en/). Restrictions may apply regarding the availability of these data, which were originally used under specific approvals for the current study and are therefore not publicly available. Access can only be given after approval by REK under the provision that the applications are consistent with the consent provided. An application form can be found on the NIPH website at https://www.fhi.no/en/studies/moba/. Specific questions regarding access to data in this study can also be directed to Dr. Per Magnus (per.magnus@fhi.no). The aggregate‐level data generated in this study are provided in the Supporting Information.
