# Supplementary material for: Parent‐of‐Origin Effects in Childhood Asthma at Seven Years of Age
Source: Genet Epidemiol. 2025 Mar 25;49(3):e70007. doi: 10.1002/gepi.70007 (PMC11937430; doi:10.1002/gepi.70007)
Supplement: Supplementary file 1 — Supporting information. [file GEPI-49-0-s001.docx]

**Supplemental File 1**

## Figure S1. Regional plots for the SNPs with parent-of-origin effects on childhood asthma
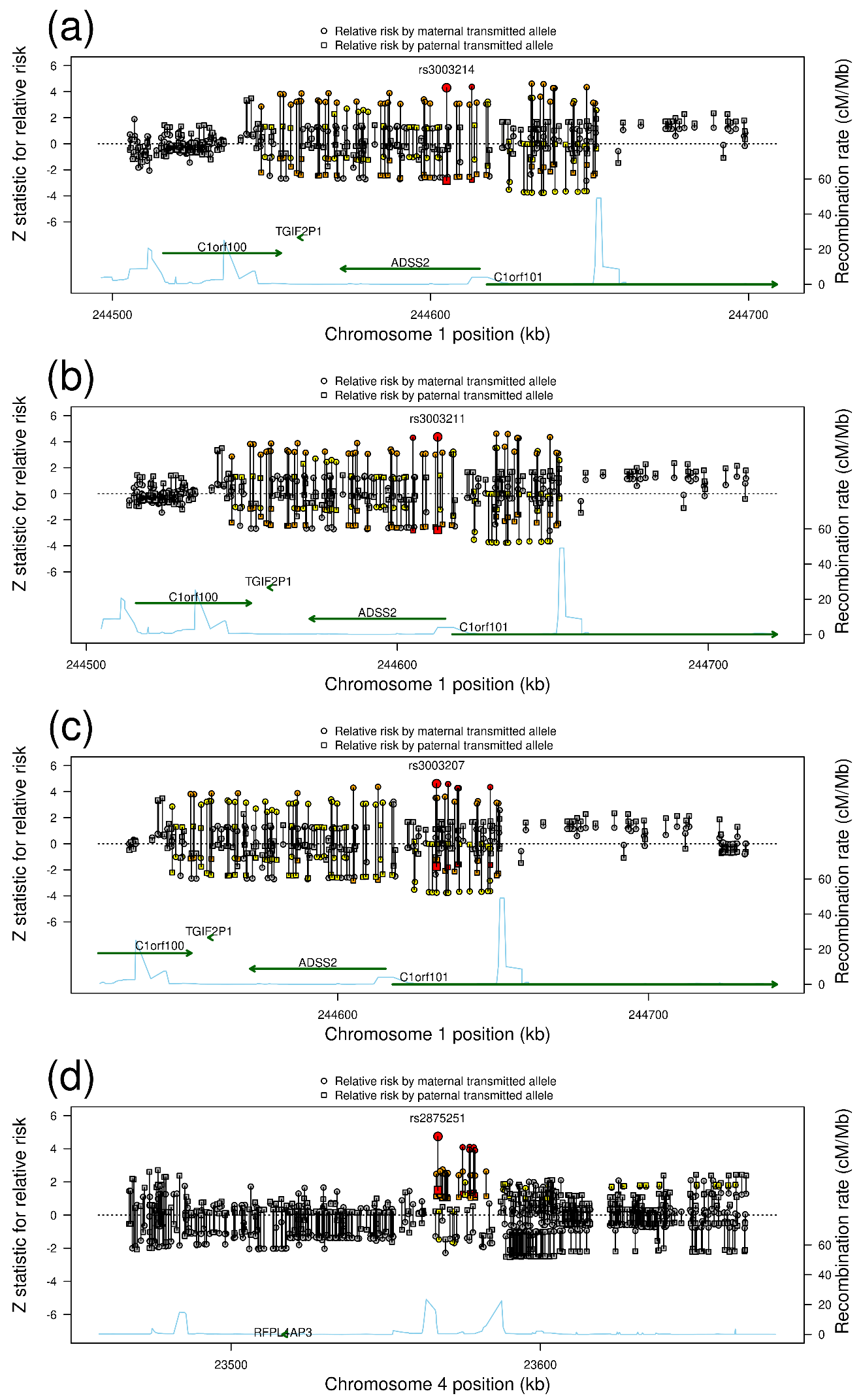


**
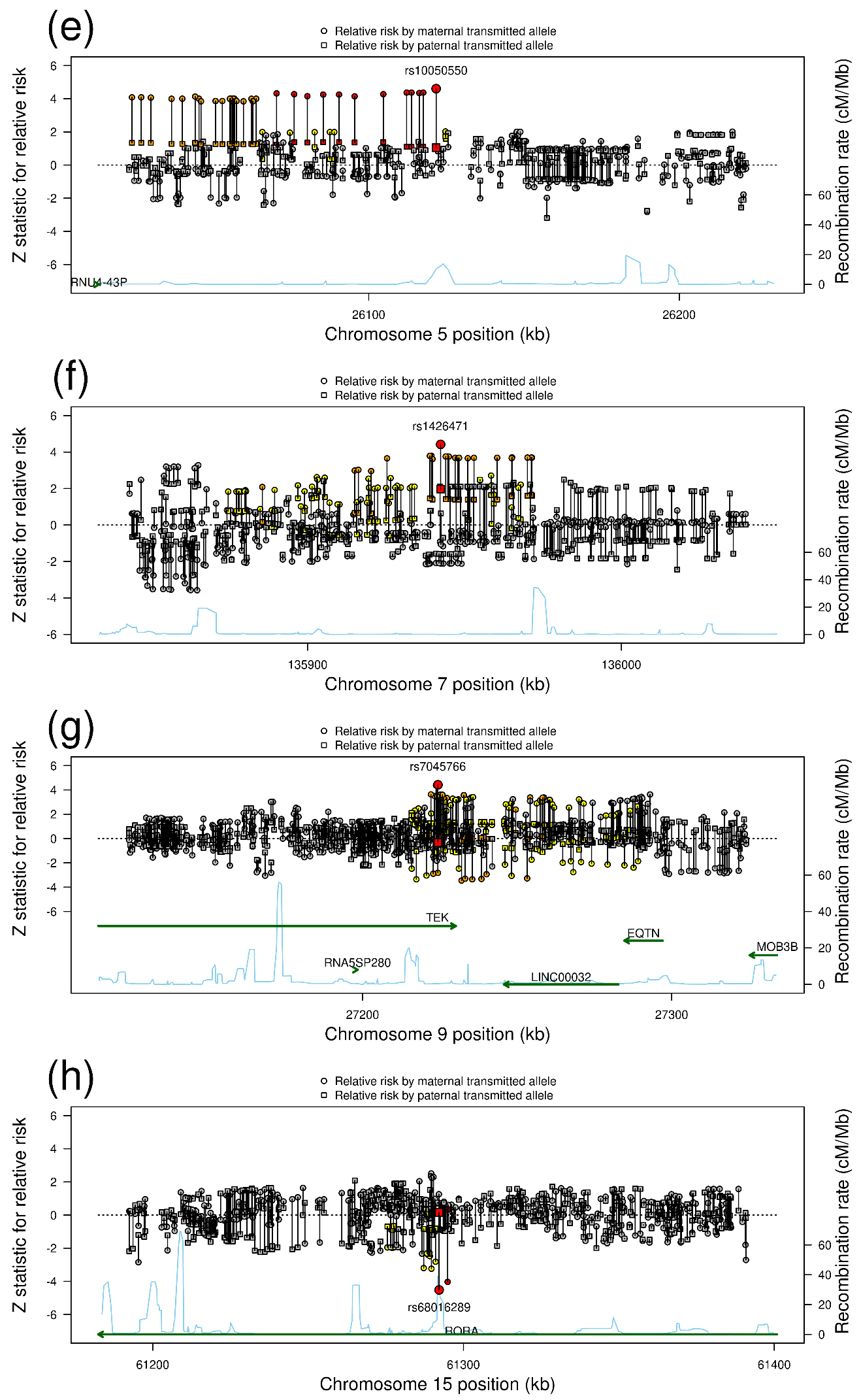

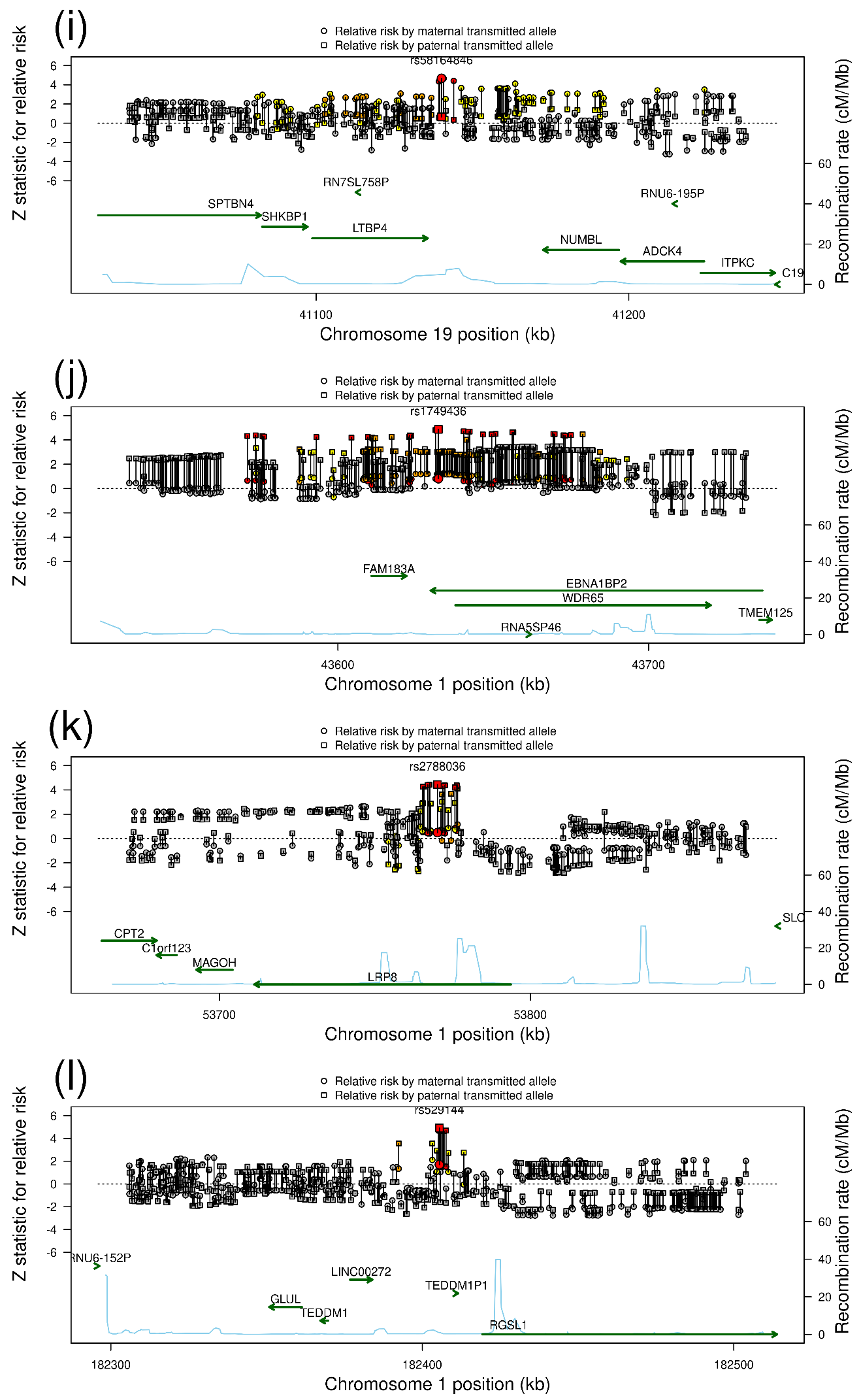

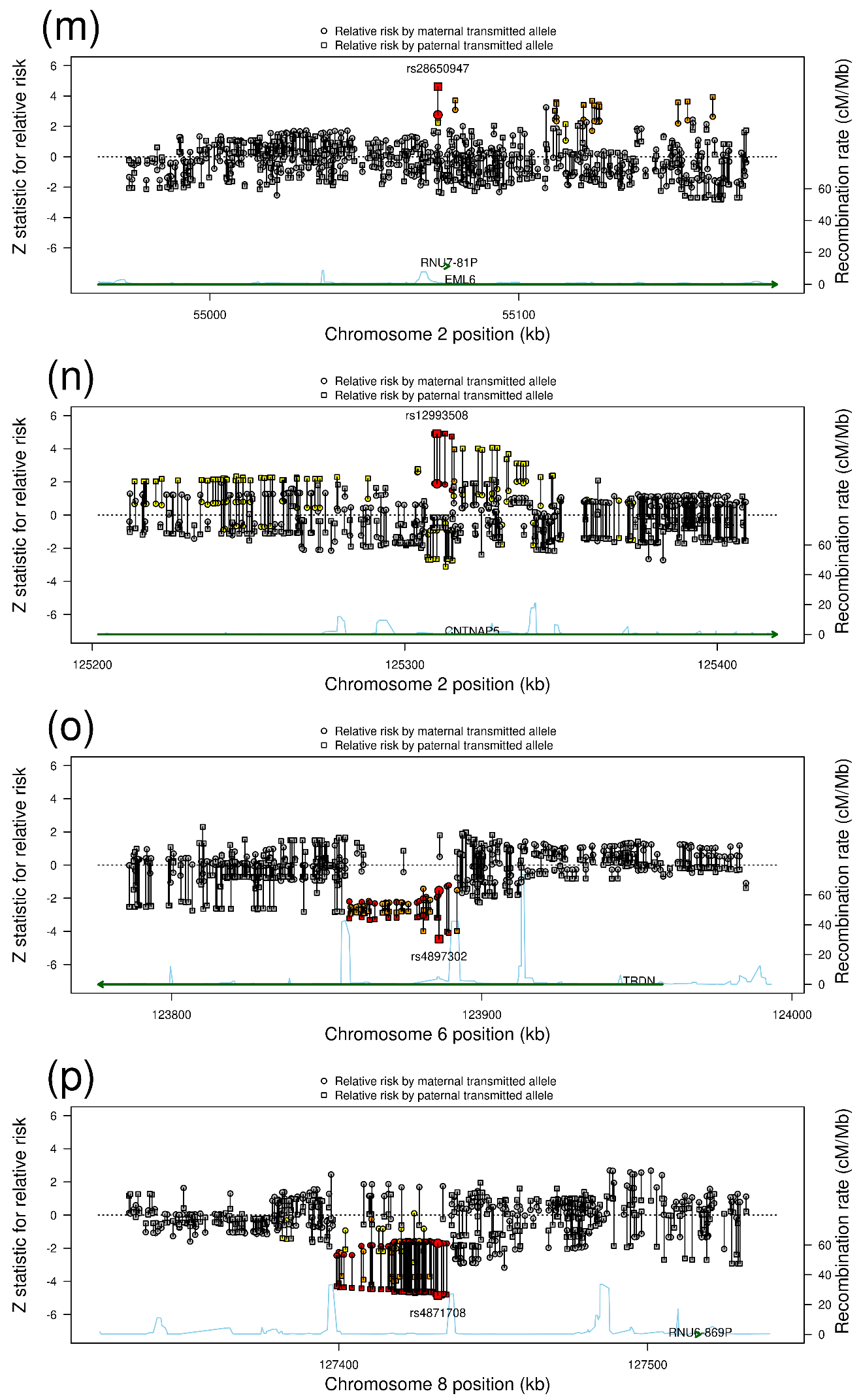

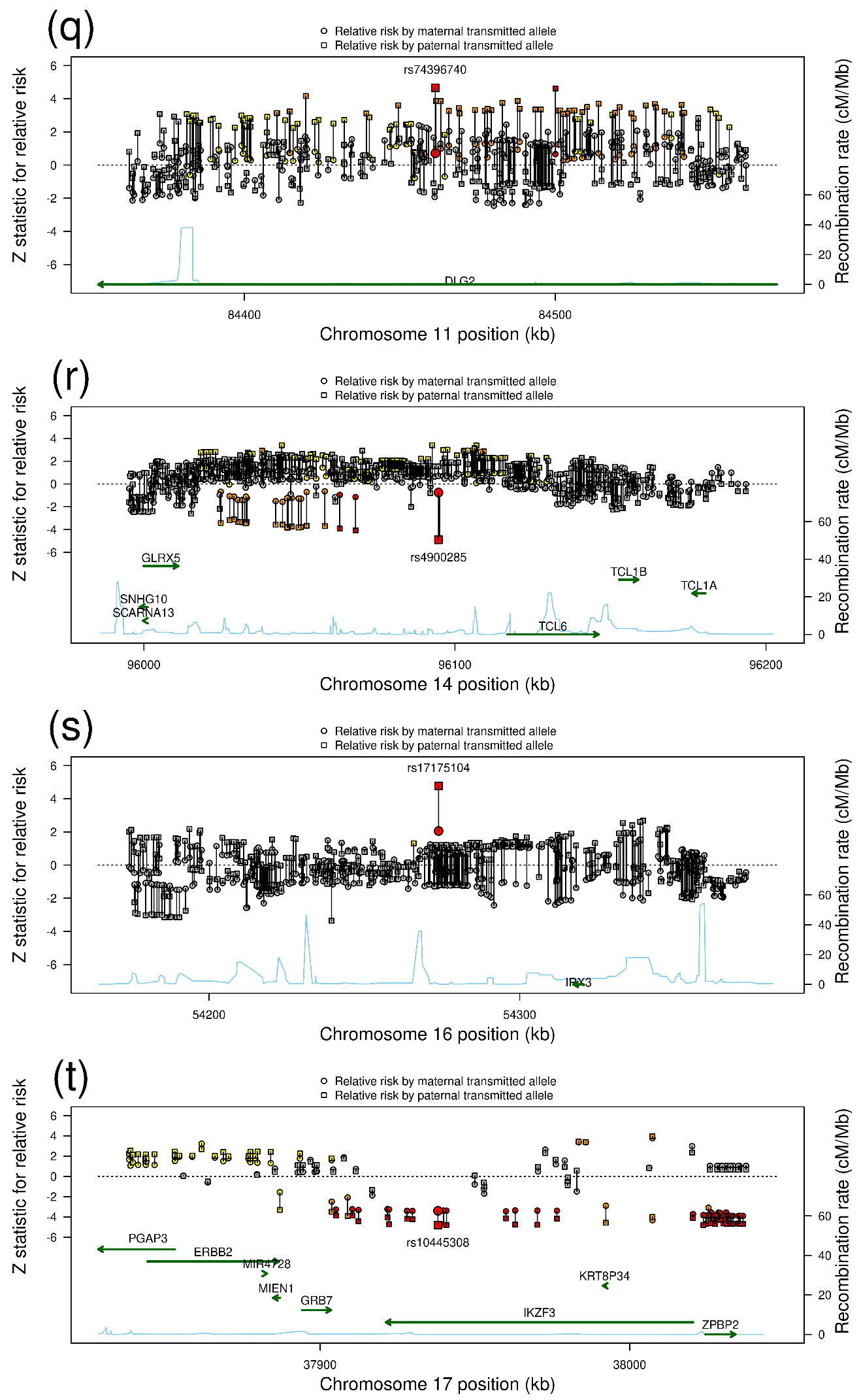

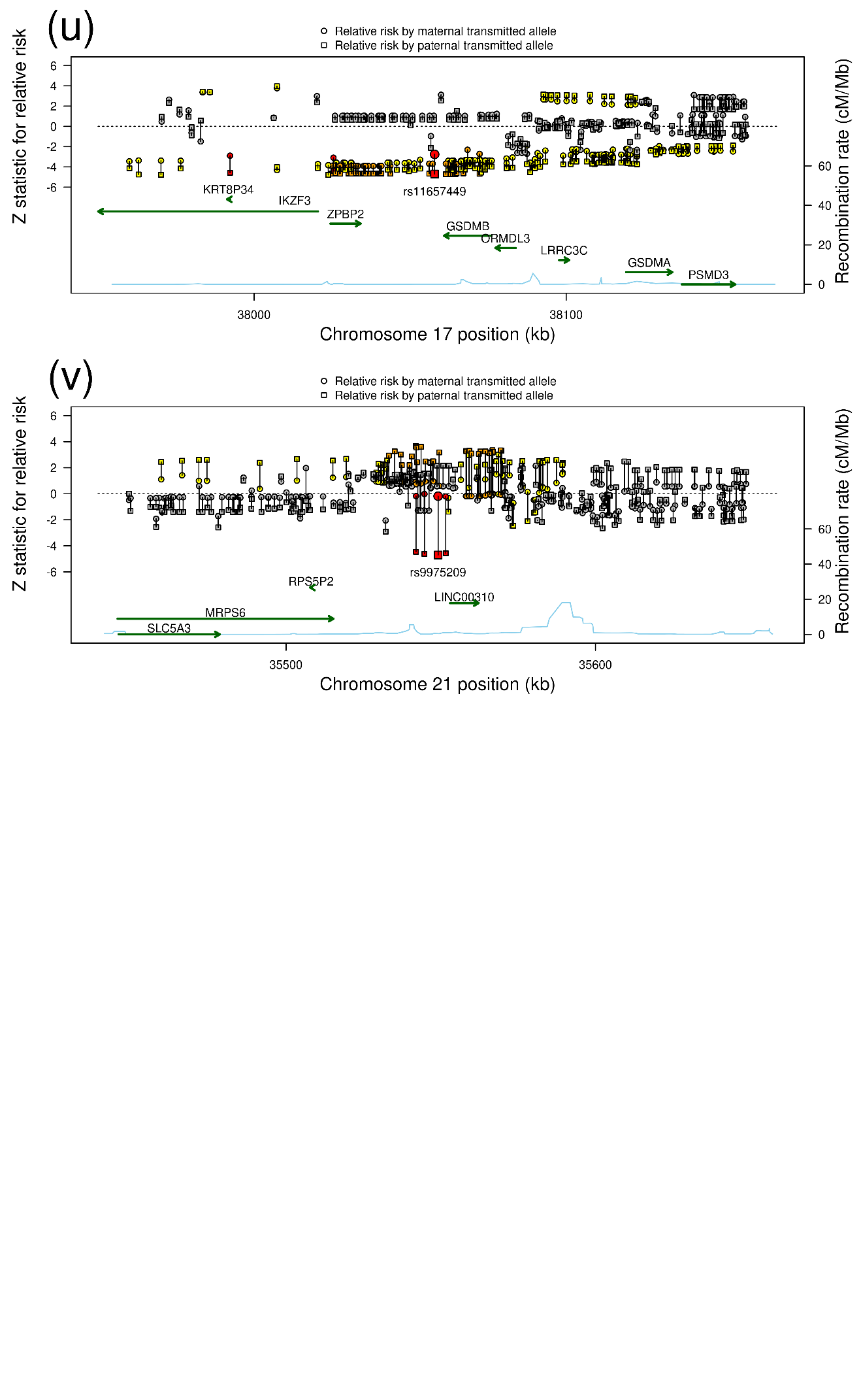
**

The x-axis is for genomic coordinates (GRCh37). The y-axis on the left-side is for the Z statistic of the relative risk, and that on the right-side is for the recombination rates that were calculated based on HapMap Phase II. A circle represents a relative risk by maternally transmitted alleles, and a square represent a relative risk by paternally transmitted alleles. A SNP of interest (in red) was placed in the center, and neighboring SNPs were displayed in different colors according to their correlation to the SNP of interest (red if R^2^≥0.8; orange if 0.5≤R^2^<0.8; yellow if 0.2≤R^2^<0.5; gray if R^2^<0.2). Gene annotations were retrieved from the Ensembl database. These plots were inspired by and recreated in the style of the LocusZoom plot (Pruim et al., 2010)

## Figure S2. Manhattan plot showing the results of maternal genetic effects in childhood asthma.

**
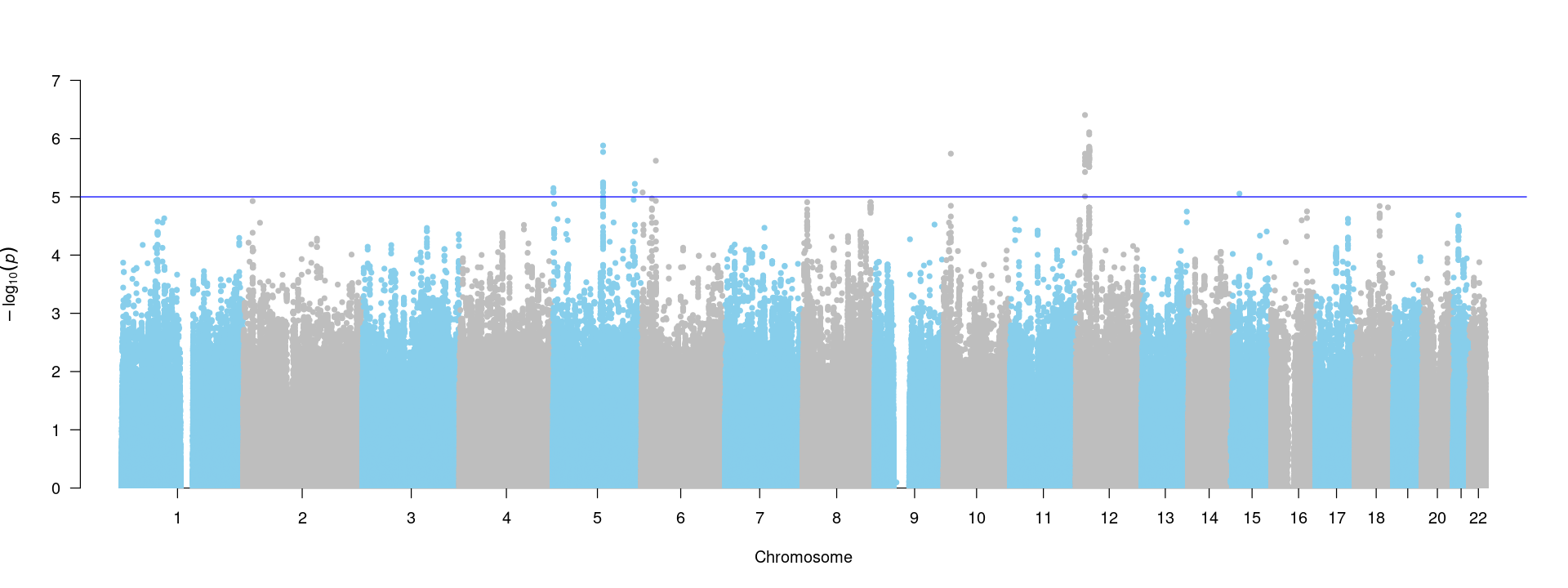
**

SNPs with a minor allele frequency>0.05 were retained in the analyses. The blue line refers to $-log \left( 1E-05 \right)=5$.

## Figure S3. Manhattan plot showing the results of fetal genetic effects in childhood asthma.


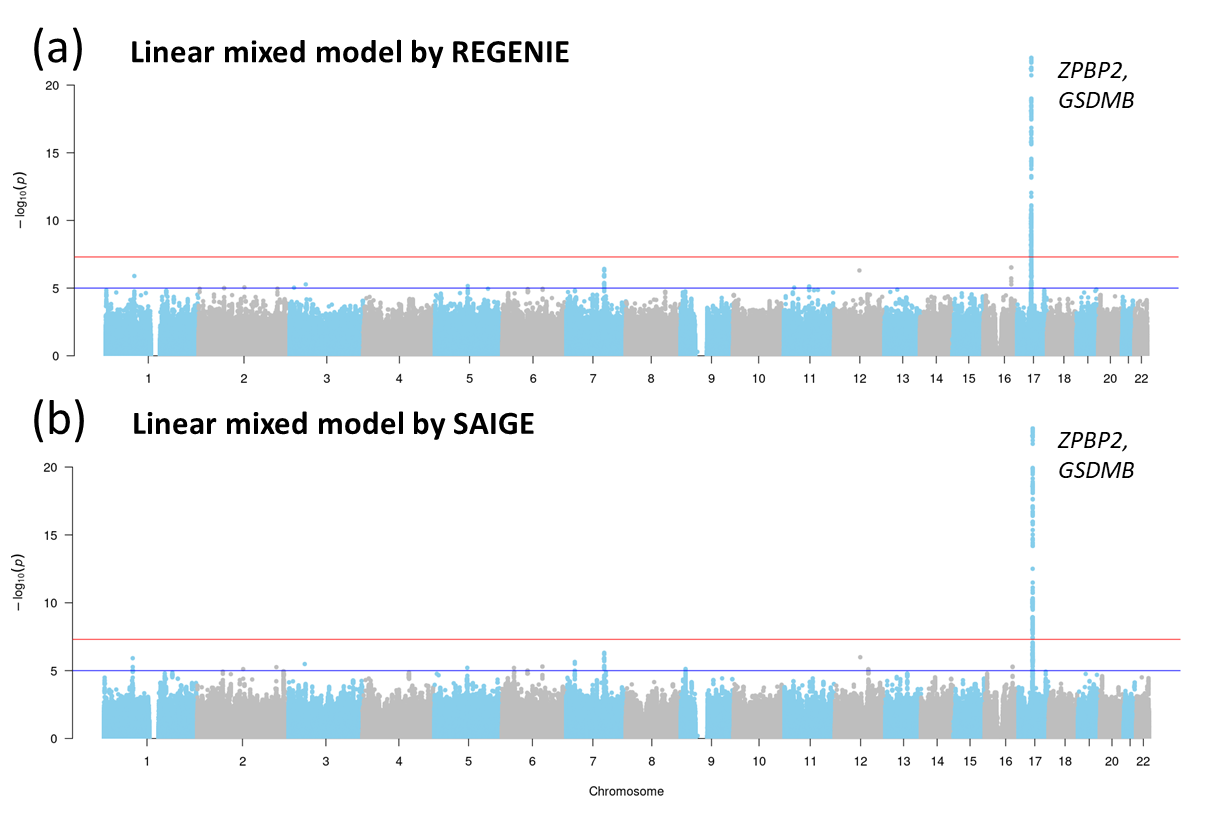


Manhattan plots that show genome-wide associations between fetal genetic loci and childhood asthma at seven years. (a) Linear mixed model was fitted using the REGENIE software (Mbatchou et al., 2021). The saddle point approximation was chosen to address the imbalance between the number of cases and controls. (b) Linear mixed model was fitted using the SAIGE R package (Zhou et al., 2018). Again, the saddle point approximation was chosen to address the imbalance between the number of cases and controls. SNPs with a minor allele frequency>0.05 were retained in the analyses. The blue horizontal line refers to $-\log_{10} \left( 10E-05 \right)=5$, whereas the red line refers to $-\log_{10} \left( 10E-08 \right)=8$.

## Table S1. SNPs with parent-of-origin effects in childhood asthma (boys).

| **SNP^a^** | **CHR** | **POS^b^** | **A1^c^** | **A2^d^** | **MAF^e^** | **HWE P-value^f^** | **Gene Names^g^** | **RRcm  (95% CI)^h^** | **RRcm  p-value^h^** | **RRcf  (95% CI)^i^** | **RRcf  p-value^i^** | **RRcm/RRcf  (95% CI)^j^** | **RRcm/RRcf  p-value^j^** |
| --- | --- | --- | --- | --- | --- | --- | --- | --- | --- | --- | --- | --- | --- |
| rs1749436 | 1 | 43632353 | C | G | 0.416 | 0.810 | *EBNA1BP2* | 1.15 (0.96, 1.38) | 1.33E-01 | 1.44 (1.21, 1.71) | 2.67E-05 | 0.8 (0.64, 0.99) | 4.17E-02 |
| rs2788036 | 1 | 53770124 | T | C | 0.367 | 0.456 | *LRP8* | 0.99 (0.82, 1.2) | 9.23E-01 | 1.27 (1.07, 1.51) | 7.29E-03 | 0.78 (0.62, 0.99) | 3.67E-02 |
| rs529144 | 1 | 182405493 | G | T | 0.160 | 0.561 |  | 1.32 (1.05, 1.65) | 1.59E-02 | 1.56 (1.27, 1.93) | 2.58E-05 | 0.85 (0.66, 1.08) | 1.76E-01 |
| rs3003214* | 1 | 244605036 | G | A | 0.324 | 0.042 | *ADSS* | 1.36 (1.13, 1.64) | 1.11E-03 | 0.78 (0.65, 0.94) | 9.93E-03 | 1.75 (1.38, 2.21) | 3.02E-06 |
| rs3003211* | 1 | 244612984 | T | C | 0.325 | 0.048 | *ADSS* | 1.36 (1.13, 1.64) | 1.20E-03 | 0.78 (0.65, 0.95) | 1.07E-02 | 1.74 (1.37, 2.19) | 3.63E-06 |
| rs3003207* | 1 | 244631821 | C | T | 0.396 | 0.102 | *C1orf101* | 1.48 (1.23, 1.78) | 3.10E-05 | 0.84 (0.7, 1) | 5.27E-02 | 1.76 (1.4, 2.21) | 1.13E-06 |
| rs28650947 | 2 | 55073797 | C | A | 0.058 | 0.855 | *EML6* | 1.33 (0.96, 1.84) | 9.09E-02 | 1.5 (1.1, 2.07) | 1.09E-02 | 0.88 (0.63, 1.23) | 4.66E-01 |
| rs12993508 | 2 | 125310284 | A | T | 0.287 | 0.700 | *CNTNAP5* | 1.06 (0.87, 1.29) | 5.61E-01 | 1.27 (1.06, 1.52) | 9.68E-03 | 0.84 (0.67, 1.05) | 1.27E-01 |
| rs2875251* | 4 | 23566880 | T | C | 0.208 | 0.702 | **PPARGC1A* | 1.34 (1.09, 1.64) | 4.78E-03 | 1.08 (0.88, 1.32) | 4.60E-01 | 1.24 (0.98, 1.56) | 6.77E-02 |
| rs10050550* | 5 | 26121701 | G | C | 0.068 | 0.404 | **CDH9* | 1.7 (1.27, 2.28) | 3.39E-04 | 1.18 (0.87, 1.62) | 2.93E-01 | 1.45 (1.04, 1.98) | 2.46E-02 |
| rs4897302 | 6 | 123886231 | C | T | 0.437 | 0.107 | *TRDN* | 0.92 (0.76, 1.11) | 3.89E-01 | 0.73 (0.61, 0.87) | 4.11E-04 | 1.26 (1, 1.58) | 4.72E-02 |
| rs1426471* | 7 | 135942399 | C | T | 0.125 | 0.719 |  | 1.58 (1.24, 1.99) | 1.32E-04 | 1.21 (0.95, 1.53) | 1.13E-01 | 1.31 (1, 1.69) | 4.53E-02 |
| rs4871708 | 8 | 127432057 | G | T | 0.214 | 0.823 |  | 0.8 (0.64, 0.99) | 4.10E-02 | 0.72 (0.58, 0.9) | 2.76E-03 | 1.11 (0.84, 1.44) | 4.64E-01 |
| rs7045766* | 9 | 27224358 | T | A | 0.403 | 0.896 | *TEK* | 1.36 (1.14, 1.63) | 8.31E-04 | 0.9 (0.76, 1.07) | 2.37E-01 | 1.51 (1.21, 1.88) | 2.30E-04 |
| rs74396740 | 11 | 84461408 | T | C | 0.079 | 0.192 | *DLG2* | 0.91 (0.66, 1.25) | 5.44E-01 | 1.47 (1.12, 1.94) | 5.68E-03 | 0.62 (0.44, 0.85) | 3.72E-03 |
| rs4900285 | 14 | 96094706 | C | T | 0.499 | 0.753 |  | 0.96 (0.79, 1.15) | 6.37E-01 | 0.74 (0.62, 0.88) | 5.50E-04 | 1.29 (1.03, 1.61) | 2.68E-02 |
| rs68016289* | 15 | 61292152 | T | C | 0.139 | 0.748 | *RORA* | 0.63 (0.47, 0.84) | 1.37E-03 | 0.95 (0.75, 1.21) | 6.69E-01 | 0.66 (0.48, 0.92) | 1.21E-02 |
| rs17175104 | 16 | 54273895 | G | A | 0.066 | 0.039 | **IRX3* | 1.24 (0.91, 1.72) | 1.77E-01 | 1.53 (1.14, 2.08) | 4.67E-03 | 0.81 (0.58, 1.13) | 2.14E-01 |
| rs10445308 | 17 | 37938047 | C | T | 0.498 | 0.820 | *IKZF3* | 0.79 (0.65, 0.94) | 1.07E-02 | 0.72 (0.61, 0.86) | 1.63E-04 | 1.09 (0.86, 1.36) | 4.74E-01 |
| rs11657449 | 17 | 38057841 | G | C | 0.330 | 0.226 | **GSDMB* | 0.81 (0.66, 0.98) | 3.34E-02 | 0.67 (0.55, 0.81) | 4.87E-05 | 1.2 (0.93, 1.54) | 1.49E-01 |
| rs58164846* | 19 | 41140115 | G | A | 0.195 | 0.023 | **LTBP4* | 1.39 (1.13, 1.72) | 1.76E-03 | 1 (0.81, 1.23) | 9.81E-01 | 1.39 (1.09, 1.79) | 7.68E-03 |
| rs9975209 | 21 | 35549135 | C | T | 0.520 | 0.620 |  | 0.93 (0.76, 1.11) | 4.02E-01 | 0.78 (0.66, 0.92) | 3.76E-03 | 1.19 (0.94, 1.49) | 1.44E-01 |

^a^ SNPs with stronger effects of maternally inherited alleles than that of paternally inherited alleles are marked with asterisk.

^b^ Nucleotide positions are according to the Genome Reference Consortium Human Build 37 (GRCh37).

^c^ Reference allele.

^d^ Effect allele.

^e^ Minor allele frequency.

^f^ Hardy–Weinberg equilibrium test.

^g^ Gene names were retrieved from the Ensembl GRCh37. When a SNP is not located within a gene, the gene nearest to the SNP is listed instead.

^h^ Relative risk of childhood asthma due to maternally inherited alleles.

^i^ Relative risk of childhood asthma due to paternally inherited alleles.

^j^ Ratio of the relative risks due to the maternally and paternally inherited alleles.

## Table S2. SNPs with parent-of-origin effects in childhood asthma (girls).

| **SNP^a^** | **CHR** | **POS^b^** | **A1^c^** | **A2^d^** | **MAF^e^** | **HWE P-value^f^** | **Gene Names^g^** | **RRcm  (95% CI)^h^** | **RRcm  p-value^h^** | **RRcf  (95% CI)^i^** | **RRcf  p-value^i^** | **RRcm/RRcf  (95% CI)^j^** | **RRcm/RRcf  p-value^j^** |
| --- | --- | --- | --- | --- | --- | --- | --- | --- | --- | --- | --- | --- | --- |
| rs1749436 | 1 | 43632353 | C | G | 0.411 | 0.050 | *EBNA1BP2* | 0.92 (0.72, 1.16) | 4.91E-01 | 1.31 (1.06, 1.63) | 1.11E-02 | 0.7 (0.52, 0.93) | 1.66E-02 |
| rs2788036 | 1 | 53770124 | T | C | 0.348 | 0.589 | *LRP8* | 1.11 (0.88, 1.41) | 3.59E-01 | 1.5 (1.22, 1.86) | 1.78E-04 | 0.74 (0.56, 0.97) | 3.26E-02 |
| rs529144 | 1 | 182405493 | G | T | 0.188 | 0.437 |  | 0.97 (0.73, 1.28) | 8.33E-01 | 1.39 (1.08, 1.78) | 8.87E-03 | 0.7 (0.51, 0.95) | 2.27E-02 |
| rs3003214* | 1 | 244605036 | G | A | 0.324 | 0.599 | *ADSS* | 1.39 (1.11, 1.74) | 3.61E-03 | 0.86 (0.68, 1.08) | 1.93E-01 | 1.62 (1.22, 2.14) | 7.20E-04 |
| rs3003211* | 1 | 244612984 | T | C | 0.326 | 0.596 | *ADSS* | 1.4 (1.12, 1.75) | 3.07E-03 | 0.87 (0.69, 1.09) | 2.13E-01 | 1.61 (1.23, 2.14) | 6.98E-04 |
| rs3003207* | 1 | 244631821 | C | T | 0.398 | 0.974 | *C1orf101* | 1.29 (1.03, 1.62) | 2.55E-02 | 0.96 (0.77, 1.19) | 6.87E-01 | 1.35 (1.02, 1.78) | 3.49E-02 |
| rs28650947 | 2 | 55073797 | C | A | 0.052 | 0.835 | *EML6* | 1.6 (1.07, 2.41) | 2.16E-02 | 2.19 (1.52, 3.23) | 3.60E-05 | 0.73 (0.49, 1.07) | 1.09E-01 |
| rs12993508 | 2 | 125310284 | A | T | 0.234 | 0.724 | *CNTNAP5* | 1.34 (1.05, 1.71) | 1.88E-02 | 1.7 (1.36, 2.14) | 2.83E-06 | 0.79 (0.6, 1.02) | 7.78E-02 |
| rs2875251* | 4 | 23566880 | T | C | 0.198 | 0.496 | **PPARGC1A* | 1.66 (1.3, 2.13) | 4.80E-05 | 1.2 (0.93, 1.54) | 1.55E-01 | 1.38 (1.05, 1.85) | 2.39E-02 |
| rs10050550* | 5 | 26121701 | G | C | 0.066 | 0.758 | **CDH9* | 1.69 (1.18, 2.43) | 4.04E-03 | 1.07 (0.72, 1.6) | 7.22E-01 | 1.58 (1.04, 2.36) | 2.93E-02 |
| rs4897302 | 6 | 123886231 | C | T | 0.445 | 0.202 | *TRDN* | 0.85 (0.68, 1.07) | 1.65E-01 | 0.74 (0.59, 0.92) | 5.74E-03 | 1.16 (0.87, 1.52) | 3.08E-01 |
| rs1426471* | 7 | 135942399 | C | T | 0.131 | 0.171 |  | 1.39 (1.04, 1.85) | 2.31E-02 | 1.18 (0.89, 1.58) | 2.51E-01 | 1.18 (0.85, 1.61) | 3.15E-01 |
| rs4871708 | 8 | 127432057 | G | T | 0.209 | 0.925 |  | 0.98 (0.75, 1.26) | 8.63E-01 | 0.55 (0.41, 0.73) | 4.76E-05 | 1.79 (1.27, 2.51) | 8.97E-04 |
| rs7045766* | 9 | 27224358 | T | A | 0.394 | 0.247 | *TEK* | 1.39 (1.12, 1.75) | 3.29E-03 | 1.1 (0.9, 1.36) | 3.53E-01 | 1.27 (0.97, 1.64) | 8.51E-02 |
| rs74396740 | 11 | 84461408 | T | C | 0.061 | 0.336 | *DLG2* | 1.46 (0.99, 2.17) | 5.50E-02 | 2.05 (1.44, 2.95) | 6.91E-05 | 0.71 (0.48, 1.04) | 8.05E-02 |
| rs4900285 | 14 | 96094706 | C | T | 0.509 | 0.290 |  | 0.94 (0.75, 1.16) | 5.44E-01 | 0.69 (0.56, 0.85) | 4.53E-04 | 1.36 (1.04, 1.77) | 2.36E-02 |
| rs68016289* | 15 | 61292152 | T | C | 0.138 | 0.550 | *RORA* | 0.56 (0.39, 0.8) | 1.53E-03 | 1.13 (0.85, 1.51) | 4.05E-01 | 0.49 (0.33, 0.74) | 5.25E-04 |
| rs17175104 | 16 | 54273895 | G | A | 0.059 | 0.628 | **IRX3* | 1.39 (0.93, 2.08) | 1.05E-01 | 2.1 (1.47, 3.04) | 5.27E-05 | 0.66 (0.45, 0.97) | 3.68E-02 |
| rs10445308 | 17 | 37938047 | C | T | 0.475 | 0.431 | *IKZF3* | 0.77 (0.61, 0.96) | 2.10E-02 | 0.72 (0.58, 0.89) | 2.54E-03 | 1.06 (0.81, 1.39) | 6.70E-01 |
| rs11657449 | 17 | 38057841 | G | C | 0.308 | 0.810 | **GSDMB* | 0.81 (0.63, 1.02) | 7.52E-02 | 0.74 (0.59, 0.94) | 1.36E-02 | 1.09 (0.8, 1.45) | 5.94E-01 |
| rs58164846* | 19 | 41140115 | G | A | 0.185 | 0.175 | **LTBP4* | 1.56 (1.22, 2.01) | 4.42E-04 | 1.15 (0.89, 1.49) | 2.79E-01 | 1.36 (1.01, 1.81) | 3.82E-02 |
| rs9975209 | 21 | 35549135 | C | T | 0.514 | 0.678 |  | 1.07 (0.86, 1.33) | 5.39E-01 | 0.66 (0.53, 0.81) | 1.00E-04 | 1.63 (1.24, 2.12) | 3.58E-04 |

^a^ SNPs with stronger effects of maternally inherited alleles than that of paternally inherited alleles are marked with asterisk.

^b^ Nucleotide positions are according to the Genome Reference Consortium Human Build 37 (GRCh37).

^c^ Reference allele.

^d^ Effect allele.

^e^ Minor allele frequency.

^f^ Hardy–Weinberg equilibrium test.

^g^ Gene names were retrieved from the Ensembl GRCh37. When a SNP is not located within a gene, the gene nearest to the SNP is listed instead.

^h^ Relative risk of childhood asthma due to maternally inherited alleles.

^i^ Relative risk of childhood asthma due to paternally inherited alleles.

^j^ Ratio of the relative risks due to the maternally and paternally inherited alleles.

## Table S3. SNPs with parent-of-origin effects in childhood asthma (including one child from each triad).

| **SNP^a^** | **CHR** | **POS^b^** | **A1^c^** | **A2^d^** | **MAF^e^** | **HWE P-value^f^** | **Gene Names^g^** | **RRcm  (95% CI)^h^** | **RRcm  p-value^h^** | **RRcf  (95% CI)^i^** | **RRcf  p-value^i^** | **RRcm/RRcf  (95% CI)^j^** | **RRcm/RRcf  p-value^j^** |
| --- | --- | --- | --- | --- | --- | --- | --- | --- | --- | --- | --- | --- | --- |
| rs1749436 | 1 | 43632353 | C | G | 0.414 | 0.362 | *EBNA1BP2* | 1.06 (0.91, 1.22) | 4.53E-01 | 1.37 (1.2, 1.57) | 3.36E-06 | 0.77 (0.65, 0.92) | 3.68E-03 |
| rs2788036 | 1 | 53770124 | T | C | 0.360 | 0.240 | *LRP8* | 1.03 (0.89, 1.2) | 7.01E-01 | 1.34 (1.17, 1.54) | 1.96E-05 | 0.77 (0.64, 0.92) | 3.62E-03 |
| rs529144 | 1 | 182405493 | G | T | 0.171 | 0.323 |  | 1.16 (0.97, 1.38) | 1.09E-01 | 1.49 (1.27, 1.75) | 1.03E-06 | 0.78 (0.64, 0.94) | 1.06E-02 |
| rs3003214* | 1 | 244605036 | G | A | 0.322 | 0.227 | *ADSS* | 1.38 (1.2, 1.6) | 1.18E-05 | 0.82 (0.71, 0.95) | 7.59E-03 | 1.68 (1.41, 2.02) | 1.54E-08 |
| rs3003211* | 1 | 244612984 | T | C | 0.323 | 0.245 | *ADSS* | 1.38 (1.2, 1.6) | 1.04E-05 | 0.82 (0.71, 0.95) | 9.05E-03 | 1.68 (1.4, 2.02) | 1.69E-08 |
| rs3003207* | 1 | 244631821 | C | T | 0.394 | 0.175 | *C1orf101* | 1.4 (1.21, 1.62) | 4.29E-06 | 0.89 (0.78, 1.03) | 1.12E-01 | 1.57 (1.31, 1.87) | 7.66E-07 |
| rs28650947 | 2 | 55073797 | C | A | 0.056 | 0.668 | *EML6* | 1.41 (1.1, 1.83) | 8.03E-03 | 1.74 (1.37, 2.24) | 5.93E-06 | 0.81 (0.62, 1.04) | 1.05E-01 |
| rs12993508 | 2 | 125310284 | A | T | 0.267 | 0.613 | *CNTNAP5* | 1.16 (0.99, 1.35) | 6.79E-02 | 1.42 (1.23, 1.63) | 1.18E-06 | 0.82 (0.68, 0.97) | 2.22E-02 |
| rs2875251* | 4 | 23566880 | T | C | 0.207 | 0.403 | **PPARGC1A* | 1.41 (1.21, 1.66) | 1.48E-05 | 1.12 (0.96, 1.31) | 1.57E-01 | 1.26 (1.05, 1.51) | 1.14E-02 |
| rs10050550* | 5 | 26121701 | G | C | 0.067 | 0.417 | **CDH9* | 1.72 (1.37, 2.17) | 2.80E-06 | 1.17 (0.91, 1.5) | 2.12E-01 | 1.48 (1.14, 1.89) | 2.57E-03 |
| rs4897302 | 6 | 123886231 | C | T | 0.442 | 0.765 | *TRDN* | 0.88 (0.76, 1.02) | 9.01E-02 | 0.74 (0.64, 0.85) | 1.30E-05 | 1.2 (1, 1.42) | 4.96E-02 |
| rs1426471* | 7 | 135942399 | C | T | 0.127 | 0.733 |  | 1.5 (1.25, 1.8) | 1.34E-05 | 1.22 (1.01, 1.46) | 3.67E-02 | 1.24 (1, 1.51) | 4.19E-02 |
| rs4871708 | 8 | 127432057 | G | T | 0.213 | 0.826 |  | 0.86 (0.73, 1.02) | 8.49E-02 | 0.65 (0.54, 0.77) | 1.05E-06 | 1.33 (1.07, 1.64) | 8.03E-03 |
| rs7045766* | 9 | 27224358 | T | A | 0.400 | 0.431 | *TEK* | 1.36 (1.18, 1.57) | 1.77E-05 | 0.96 (0.84, 1.1) | 5.81E-01 | 1.42 (1.19, 1.68) | 6.67E-05 |
| rs74396740 | 11 | 84461408 | T | C | 0.072 | 0.961 | *DLG2* | 1.08 (0.84, 1.38) | 5.39E-01 | 1.66 (1.34, 2.07) | 4.56E-06 | 0.65 (0.51, 0.83) | 7.57E-04 |
| rs4900285 | 14 | 96094706 | C | T | 0.503 | 0.466 |  | 0.95 (0.82, 1.09) | 4.62E-01 | 0.71 (0.62, 0.82) | 6.67E-07 | 1.33 (1.12, 1.58) | 1.34E-03 |
| rs68016289* | 15 | 61292152 | T | C | 0.140 | 0.839 | *RORA* | 0.59 (0.47, 0.75) | 6.28E-06 | 1.01 (0.84, 1.22) | 9.44E-01 | 0.59 (0.46, 0.76) | 4.56E-05 |
| rs17175104 | 16 | 54273895 | G | A | 0.064 | 0.196 | **IRX3* | 1.31 (1.02, 1.68) | 3.49E-02 | 1.72 (1.37, 2.18) | 3.69E-06 | 0.76 (0.59, 0.98) | 3.22E-02 |
| rs10445308 | 17 | 37938047 | C | T | 0.490 | 0.840 | *IKZF3* | 0.78 (0.68, 0.9) | 9.42E-04 | 0.73 (0.63, 0.83) | 2.68E-06 | 1.08 (0.9, 1.29) | 4.08E-01 |
| rs11657449 | 17 | 38057841 | G | C | 0.321 | 0.276 | **GSDMB* | 0.82 (0.7, 0.96) | 1.09E-02 | 0.71 (0.61, 0.82) | 6.02E-06 | 1.16 (0.95, 1.4) | 1.32E-01 |
| rs58164846* | 19 | 41140115 | G | A | 0.192 | 0.017 | **LTBP4* | 1.44 (1.23, 1.7) | 6.86E-06 | 1.06 (0.9, 1.25) | 5.08E-01 | 1.37 (1.13, 1.65) | 1.10E-03 |
| rs9975209 | 21 | 35549135 | C | T | 0.517 | 0.556 |  | 0.99 (0.86, 1.14) | 8.81E-01 | 0.73 (0.64, 0.83) | 2.55E-06 | 1.36 (1.14, 1.62) | 5.64E-04 |

^a^ SNPs with stronger effects of maternally inherited alleles than that of paternally inherited alleles are marked with asterisk.

^b^ Nucleotide positions are according to the Genome Reference Consortium Human Build 37 (GRCh37).

^c^ Reference allele.

^d^ Effect allele.

^e^ Minor allele frequency.

^f^ Hardy–Weinberg equilibrium test.

^g^ Gene names were retrieved from the Ensembl GRCh37. When a SNP is not located within a gene, the gene nearest to the SNP is listed instead.

^h^ Relative risk of childhood asthma due to maternally inherited alleles.

^i^ Relative risk of childhood asthma due to paternally inherited alleles.

^j^ Ratio of the relative risks due to the maternally and paternally inherited alleles.

## Table S4. Top 20 SNPs with fetal genetic effects in childhood asthma.

| **SNP^a^** | **CHR** | **Position^b^** |  |  |  | **Linear mixed model  by REGENIE** | | | |  | **Linear mixed model  by SAIGE** | | | |
| --- | --- | --- | --- | --- | --- | --- | --- | --- | --- | --- | --- | --- | --- | --- |
|  |  |  | **Reference Allele** | **Effect Allele** | **MAF^c^** | **Beta** | **SE** | **T** | **P** |  | **Beta** | **SE** | **CHISQ** | **P** |
| rs8069176 | 17 | 38057197 | G | A | 0.476 | -0.357 | 0.036 | -282.671 | 1.40E-23 |  | -0.356 | 0.036 | 96.332 | 9.71E-23 |
| rs11651596 | 17 | 38056116 | T | C | 0.476 | -0.357 | 0.036 | -282.424 | 1.53E-23 |  | -0.356 | 0.036 | 96.291 | 9.92E-23 |
| rs12949100 | 17 | 38057189 | G | A | 0.476 | -0.357 | 0.036 | -282.291 | 1.58E-23 |  | -0.356 | 0.036 | 96.201 | 1.04E-22 |
| rs4795399 | 17 | 38061439 | T | C | 0.476 | -0.357 | 0.036 | -282.295 | 1.61E-23 |  | -0.355 | 0.036 | 95.918 | 1.20E-22 |
| rs11078928 | 17 | 38064469 | T | C | 0.475 | -0.356 | 0.036 | -281.419 | 2.15E-23 |  | -0.354 | 0.036 | 95.313 | 1.63E-22 |
| rs11078926 | 17 | 38062976 | G | A | 0.475 | -0.356 | 0.036 | -281.424 | 2.15E-23 |  | -0.354 | 0.036 | 95.311 | 1.63E-22 |
| rs11078927 | 17 | 38064405 | C | T | 0.475 | -0.356 | 0.036 | -281.381 | 2.18E-23 |  | -0.354 | 0.036 | 95.310 | 1.63E-22 |
| rs2305480 | 17 | 38062196 | G | A | 0.475 | -0.355 | 0.036 | -280.953 | 2.54E-23 |  | -0.354 | 0.036 | 94.951 | 1.95E-22 |
| rs12939832 | 17 | 38064876 | G | A | 0.475 | -0.355 | 0.036 | -280.516 | 2.90E-23 |  | -0.353 | 0.036 | 94.705 | 2.21E-22 |
| rs59716545 | 17 | 38031857 | T | G | 0.485 | -0.353 | 0.036 | -279.706 | 4.12E-23 |  | -0.350 | 0.036 | 93.060 | 5.07E-22 |
| rs12709365 | 17 | 38027400 | A | G | 0.485 | -0.353 | 0.036 | -279.421 | 4.64E-23 |  | -0.349 | 0.036 | 92.758 | 5.91E-22 |
| rs13380815 | 17 | 38027583 | A | G | 0.485 | -0.353 | 0.036 | -279.421 | 4.64E-23 |  | -0.349 | 0.036 | 92.758 | 5.91E-22 |
| rs1054609 | 17 | 38033277 | A | C | 0.485 | -0.352 | 0.036 | -279.370 | 4.73E-23 |  | -0.349 | 0.036 | 92.703 | 6.08E-22 |
| rs10852936 | 17 | 38031714 | C | T | 0.485 | -0.352 | 0.036 | -279.331 | 4.81E-23 |  | -0.349 | 0.036 | 92.698 | 6.09E-22 |
| rs11870965 | 17 | 38030205 | T | A | 0.485 | -0.352 | 0.036 | -279.331 | 4.81E-23 |  | -0.349 | 0.036 | 92.698 | 6.09E-22 |
| rs36038753 | 17 | 38035370 | G | T | 0.485 | -0.352 | 0.036 | -279.331 | 4.81E-23 |  | -0.349 | 0.036 | 92.698 | 6.09E-22 |
| rs9905959 | 17 | 38031138 | A | G | 0.485 | -0.352 | 0.036 | -279.331 | 4.81E-23 |  | -0.349 | 0.036 | 92.698 | 6.09E-22 |
| rs9907088 | 17 | 38035116 | G | A | 0.485 | -0.352 | 0.036 | -279.331 | 4.81E-23 |  | -0.349 | 0.036 | 92.698 | 6.09E-22 |
| rs9904624 | 17 | 38036586 | A | G | 0.485 | -0.352 | 0.036 | -279.330 | 4.83E-23 |  | -0.349 | 0.036 | 92.698 | 6.09E-22 |
| rs9910826 | 17 | 38035648 | A | G | 0.485 | -0.352 | 0.036 | -279.330 | 4.83E-23 |  | -0.349 | 0.036 | 92.698 | 6.09E-22 |

^a^ Located near or within *ZPBP2,* the gene name of which was retrieved from the *Ensembl* database based on the human genome version GRCh37.

^b^ Genome Reference Consortium Human Build 37.

^c^ Minor allele frequency.

**References**

Mbatchou, J., Barnard, L., Backman, J., Marcketta, A., Kosmicki, J. A., Ziyatdinov, A., . . . Marchini, J. (2021). Computationally efficient whole-genome regression for quantitative and binary traits. *Nat Genet, 53*(7), 1097-1103. doi:10.1038/s41588-021-00870-7

Pruim, R. J., Welch, R. P., Sanna, S., Teslovich, T. M., Chines, P. S., Gliedt, T. P., . . . Willer, C. J. (2010). LocusZoom: regional visualization of genome-wide association scan results. *Bioinformatics, 26*(18), 2336-2337. doi:10.1093/bioinformatics/btq419

Zhou, W., Nielsen, J. B., Fritsche, L. G., Dey, R., Gabrielsen, M. E., Wolford, B. N., . . . Lee, S. (2018). Efficiently controlling for case-control imbalance and sample relatedness in large-scale genetic association studies. *Nat Genet, 50*(9), 1335-1341. doi:10.1038/s41588-018-0184-y
