# Supplementary material for: Parent‐of‐Origin Effects in Childhood Asthma at Seven Years of Age
Source: Genet Epidemiol. 2025 Mar 25;49(3):e70007. doi: 10.1002/gepi.70007 (PMC11937430; doi:10.1002/gepi.70007)
Supplement: Supplementary file 4 — Supporting information. [file GEPI-49-0-s002.docx]

# Supplemental Materials

## Supplemental File 1.docx

This file includes i) regional plots for the SNPs with PoO effects in childhood asthma, ii) Manhattan plot showing the results of maternal genetic effects in childhood asthma, iii) Manhattan plots for the GWAS of children’s own genotypic effects and childhood asthma, iv) sensitivity analyses for the SNPs with PoO effects, and v) summary statistics for the GWAS of children’s own genotypic effects and childhood asthma.

## Supplemental File 2.csv

This file includes summary statistics for the SNPs with PoO effects in childhood asthma.

## Supplemental File 3.csv

This file includes summary statistics for the haplotypes with PoO effects in childhood asthma.
